# Radiotherapy‐Associated Cellular Senescence and EMT Alterations Contribute to Distinct Disease Relapse Patterns in Locally Advanced Cervical Cancer

**DOI:** 10.1002/advs.202412574

**Published:** 2025-02-04

**Authors:** Lei Zhang, Jun Ma, Jun Zhang, Minjie Hu, Jinlin Cheng, Bin Hu, Junjun Zhou, Di Zhou, Yongrui Bai, Xiumei Ma, Jianming Tang, Haiyan Chen, Ying Jing

**Affiliations:** ^1^ Department of Radiation Oncology Renji Hospital School of Medicine Shanghai Jiao Tong University Shanghai 200127 China; ^2^ Eye Institute Eye & ENT Hospital Shanghai Medical College Fudan University Shanghai 200031 China; ^3^ Center for Intelligent Medicine Research Greater Bay Area Institute of Precision Medicine (Guangzhou) School of Life Sciences Fudan University Guangzhou 511400 China; ^4^ State Key Laboratory of Genetic Engineering Center for Evolutionary Biology School of Life Sciences Fudan University Shanghai 200438 China; ^5^ Department of Radiation Oncology The First Hospital of Lanzhou University Lanzhou University Lanzhou 730000 China; ^6^ State Key Laboratory for Diagnosis and Treatment of Infectious Diseases National Clinical Research Center for Infectious Diseases National Medical Center for Infectious Diseases Collaborative Innovation Center for Diagnosis and Treatment of Infectious Diseases The First Affiliated Hospital Zhejiang University School of Medicine Hangzhou Zhejiang 310003 China

**Keywords:** cellular senescence, cervical squamous carcinoma, disease relapse pattern, epithelial‐mesenchymal transition, radiotherapy, single‐nucleus RNA sequencing

## Abstract

A notable number of locally advanced cervical carcinoma (LACC) patients experience local or distant disease relapse following radiotherapy. The contribution of tumor microenvironment (TME) to tumor recurrence at different sites remains unclear. Here, single‐nucleus RNA sequencing data from 28 pre‐ and on‐treatment LACC samples from patients with different disease relapse patterns is analyzed. The findings revealed opposing alterations in the expression levels of the cellular senescence pathway after radiotherapy in patients with local and distant relapses. In contrast, an increase in the expression of the epithelial‐mesenchymal transition module after radiotherapy in both relapse groups is observed. Cell–cell interactions, drug‐target expression analyses in malignant cells after radiation, and multiplex immunofluorescence of tumor tissue identified interleukin‐1 receptor type I (IL1R1) as a potential therapeutic target. It is demonstrated that combining the IL1R1 inhibitor anakinra with radiation can mitigate the effects of radiation on tumor cells. This study highlights the distinct roles of cellular senescence and EMT in tumor recurrence.

## Graphical abstract

The relapse rate for locally advanced cervical cancer (LACC) patients after radiotherapy is 20%–40%. This study shows that the cellular senescence pathway in malignant cells varies among relapse patterns, while EMT module expression is consistently elevated in all relapse patterns. Additionally, the cellular senescence status of fibroblasts ubiquitously increased following radiotherapy.

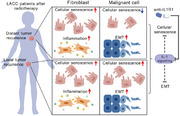

## 1 Introduction

Cervical cancer, as the third leading cause of cancer‐related death, is a prevalent and challenging malignancy among women globally.^[^ ^1^ ^]^ Despite advancements in treatment options such as surgery, radiation therapy, and chemotherapy, ≈40%–50% of all cervical cancer patients are initially diagnosed with locally advanced disease, and some lose the opportunity for surgical interventions.^[^ ^2^ ^]^ Radiotherapy is an important treatment for locally advanced cervical cancer (LACC), but the prognosis remains poor due to high recurrence rates.^[^ ^3^ ^]^ Notably, disease relapse in LACC patients following radiotherapy manifests in distinct patterns, either locally or at distant sites. Some patients experience relapse within the irradiated field, while others develop distant metastases.^[^ ^4^ ^]^ Patients with distant tumor recurrences generally have a lower overall survival rate compared to those with local recurrences.^[^ ^5^ ^]^ These diverse relapse patterns suggest that different biological processes may be at play, and they may potentially be influenced by the extent and nature of radiotherapy‐induced cellular changes, particularly within the tumor microenvironment (TME). The TME, which comprises various malignant and stromal cells, plays a crucial role in tumor progression and treatment response.^[^ ^6^ ^]^ However, the complex cellular responses to radiotherapy and the underlying mechanisms driving different relapse patterns remain poorly understood. A deeper understanding of these processes is necessary for developing potentially new therapeutic approaches and improving outcomes for LACC patients undergoing radiotherapy.

Increasing evidence suggests that radiotherapy plays a multifaceted role in treatment efficacy.^[^ ^7^ ^]^ While it effectively targets and eliminates tumor cells, emerging evidence suggests it can also induce cellular senescence, a state of permanent arrest in the cell cycle traditionally considered a deterrent to tumor progression.^[^ ^8^ ^]^ However, senescent cells can persist in tissues, secreting various factors known as the senescence‐associated secretory phenotype (SASP). SASP has been linked to promoting inflammation as well as altering the composition of the TME and potentially facilitating tumor growth in certain contexts.^[^ ^9^ ^]^ Furthermore, studies have shown that surviving tumor cells may undergo epithelial‐to‐mesenchymal transition (EMT) following radiotherapy.^[^ ^10^ ^]^ EMT, a process where epithelial cells acquire mesenchymal characteristics, is linked to increased tumor aggressiveness and the development of resistance to anti‐cancer drugs.^[^ ^11^ ^]^ This transition not only promotes cancer cell invasion and metastasis but it can also contribute to the generation of cancer stem cells, a subpopulation known for their high regenerative capacity and resistance to conventional therapies.^[^ ^12^ ^]^ It remains unclear how radiotherapy‐induced senescence and EMT contribute to different disease relapse patterns and whether these processes interact with each other, subsequently promoting tumor recurrence at different sites in an individual after radiotherapy.

Based on this background, we hypothesized that cellular senescence and EMT, potentially interact with each other, thereby creating a dynamic cellular state, which affects tumor behavior and treatment response after radiotherapy. To test this hypothesis, we conducted single‐nucleus RNA sequencing on pre‐ and on‐treatment tumor samples from LACC patients with varying relapse patterns. This approach allowed us to characterize key transitions in the TME in response to radiation. Our results provide a comprehensive overview of the TME landscape related to tumor recurrence, revealing the diverse roles of cellular senescence and EMT in LACC relapse. We also identified interleukin‐1 receptor type I (IL1R1) as a potential therapeutic target for patients who experience a disease relapse. Our findings enhance our understanding of tumor recurrence mechanisms and these may guide the development of more effective treatments for improving radiotherapy outcomes. In addition, this may lead to a reduction in the risk of disease relapse in LACC patients.

## 2 Results

### 2.1 Single‐Nucleus Analysis Revealed Transcriptional Heterogeneity in LACC Patients with Different Disease Relapse Patterns Observed after Radiotherapy

**Figure 1:**
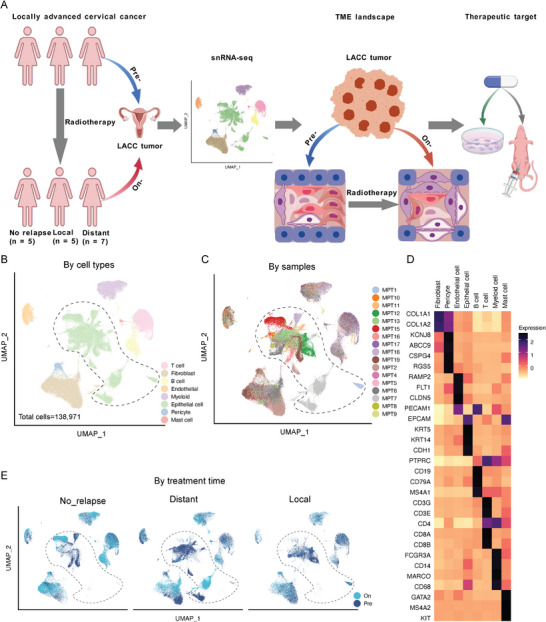
Divergent distribution of epithelial cell subclusters in tumor samples from patients with different tumor recurrent sites. A) A schematic diagram of the treatment design and sample collection process. Patients with LACC were categorized into three groups: loco‐regional, distant, and no tumor recurrences based on their relapse patterns. The tumor samples were collected at the indicated time points. Created with BioGDP.com.^[^ ^30^ ^]^ B, C) Uniform manifold approximation and projection (UMAP) plots of single‐nucleus profiles of tumors from all patients. In B, the dots are colored by major cell types, and in C, the dots are colored by individual patients. D) A heatmap displaying the expression levels of well‐known cell type marker genes for each major cell type. E) UMAP plots showing the distribution of single nuclei in all the different disease relapse groups. The dots are colored based on sample collection time. The epithelial cell clusters are outlined by dashed lines. On, on‐treatment; Pre, pre‐treatment.

We monitored the entire treatment course of patients receiving radiotherapy, following up for at least two years or when a tumor relapse occurred. Disease relapse patterns were evaluated using imaging techniques such as CT, MRI, and PET‐CT, along with histological confirmation (Figure S1, Supporting Information). Our cohort included patients with loco‐regional tumor recurrences (Local, N = 5), distant tumor recurrences (Distant, N = 7), and no tumor recurrences (No relapse, N = 5; **Figure** **1**A and Table S1, Supporting Information). We collected 28 samples, including 22 matched pre‐ and on‐treatment tumor tissues from 11 patients. These samples enabled both baseline associative analyses related to disease relapse patterns as well as longitudinal tracking of TME dynamics during therapeutic interventions. We performed single‐nucleus RNA sequencing (snRNA‐seq) on freshly frozen samples. After rigorous quality filtering, we retained 138971 high‐quality nuclei for subsequent analyses. We subsequently identified eight major cell types, including epithelial cells, fibroblasts, and endothelial cells by utilizing unsupervised clustering and canonical marker‐based annotation (Figure 1B,D). The immune and stromal cells from different samples were clustered together by cell types and this indicated minimal batch effects (Figure 1C). However, with respect to the epithelial cells, the majority of these were malignant, and they generally clustered based on their respective samples (Figure 1C). We observed an almost mutually exclusive distribution pattern of the epithelial cell clusters among the three disease relapse groups (Figure 1E), which highlights the major impact of radiotherapy on the TME that is related to disease relapse.

### 2.2 Distinct Cellular Senescent and EMT Statuses in Malignant Cells Correlated with Different Disease Relapse Patterns

**Figure 2:**
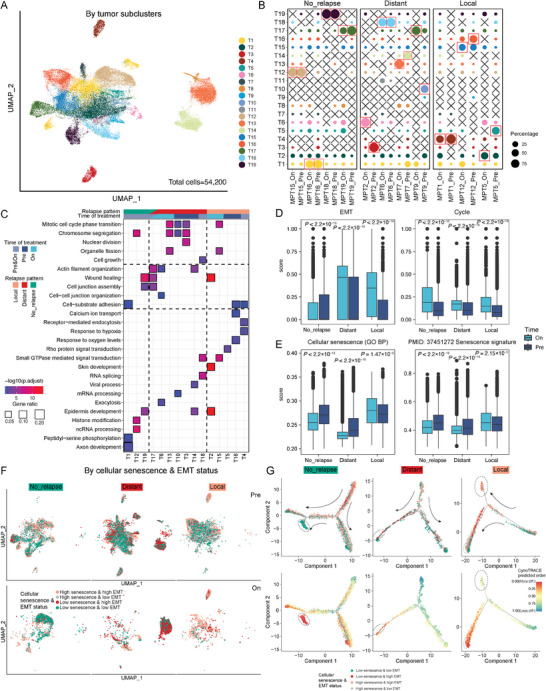
Malignant cells from different disease relapse groups exhibited distinct cellular senescence and EMT expression levels. A) Uniform manifold approximation and projection (UMAP) plot of malignant cells from all the tumor samples, showing 19 distinct subclusters. Each dot represents a single nucleus, color‐coded by the tumor cell subcluster. B) The tumor cell compositions of different subclusters in the three disease relapse patterns. The circle size represents the proportion of subclusters (among the total quality‐control‐passed malignant cells) in each sample. The circles are color‐coded by subclusters as described in panel A. C) Gene ontology biological process (GO BP) enrichment analysis for the highly expressed genes in each subcluster. Each column represents a subcluster, color‐coded by the group of samples contributing the largest proportion showing in B. D, E) Box plots showing differences in expression of cancer cell state (D) and cellular senescence (E) signatures between on‐ and pre‐treatment samples in the multiple disease relapse groups. The *P*‐values were calculated using a two‐sided Wilcoxon rank‐sum test. The center line indicates the median value, with the lower and upper hinges representing the 25th and 75th percentiles, respectively, and the whiskers denoting the 1.5 interquartile range. F) UMAP plots showing the distribution of malignant cells in the different disease relapse groups. The dots are colored based on their expression levels of cellular senescence and EMT signatures. G) Top, Monocle 2 trajectory reconstruction analysis of malignant cell differentiation in all the different disease relapse groups. Cells are color‐coded by their expression levels of cellular senescence and EMT signatures. Bottom, the same trajectory plot as above, but with cells color‐coded by their CytoTRACE score. On, on‐treatment; Pre, pre‐treatment.

We identified malignant cells by analyzing the inferred copy‐number variations (CNVs) in epithelial cells (Figure S2, Supporting Information), which were comparable to those derived from The Cancer Genome Atlas (TCGA) cervical cancer cohort and our previous study.^[^ ^13^ ^]^ The majority of the epithelial cells were found to be malignant (Figure S2, Supporting Information), and 54200 malignant cells derived from 17 patients were clustered into 19 distinct subclusters (**Figure** **2**A; Figure S3A,C, Supporting Information). In the patients who did not relapse, the largest malignant cell subclusters in pre‐ and on‐treatment tumors were identical, whereas, in most patients who experienced relapse, the subclusters were markedly different (Figure 2B; Figure S3B, Supporting Information). Pathway enrichment analysis revealed that highly expressed genes in each subcluster across the three‐relapsed pattern patient groups were enriched in different pathways related to cell growth and cell adhesion (Figure 2C).

These results prompted us to further investigate the malignant cell states associated with the divergent disease relapse patterns, focusing on radiation‐induced cellular senescence^[^ ^14^ ^]^ and EMT. We scored each malignant cell for the expression of well‐established cancer cell state gene modules,^[^ ^15^ ^]^ cellular senescence‐related signatures^[^ ^14^, ^16^ ^],^ and hallmark gene sets from MsigDB^[^ ^17^ ^]^ (Figure S4, Supporting Information). The cycle module, consisting of cell cycle genes, was expressed at higher levels in the on‐treatment samples when compared to the pre‐treatment ones (Figure 2D). Notably, the expression of both cellular senescence and complete EMT module was increased in malignant cells from on‐treatment samples relative to pre‐treatment samples in patients from the local group. In contrast, in patients with distant recurrences, the malignant cells exhibited decreased expression of cellular senescence and increased expression of EMT in on‐treatment samples when compared to pre‐treatment ones (Figure 2D,E).

We categorized malignant cells into four distinct statuses based on the expression levels of cellular senescence and EMT (Figure 2F). In the on‐treatment tumor samples, the majority of malignant cells in patients with distant recurrences were characterized as low senescence and high EMT (Low senescence & high EMT), while in patients with local recurrences, these cells were predominantly high in both senescence and EMT (high senescence & high EMT) (Figure 2F). However, in pre‐treatment tumors, the distribution of these four statuses in malignant cells was similar in all the relapse groups (Figure 2F). Trajectory analysis using Monocle 2^[^ ^18^ ^]^ and CytoTRACE^[^ ^19^ ^]^ revealed distinct differentiation paths in these groups. The malignant cells initially had a mixture of statuses and then they differentiated into a high senescence & low EMT status, a low senescence & high EMT status, and a high senescence & high EMT status in the no relapse, distant relapse, and local relapse groups, respectively (Figure 2G). In summary, our analysis indicated that there were different tumor recurrence patterns in LACC patients after radiotherapy and these were primarily associated with observed increased expression levels of the EMT module and opposite trends in cellular senescence.

### 2.3 Validation of Malignant Cell Status Changes Associated with Radiotherapy

**Figure 3:**
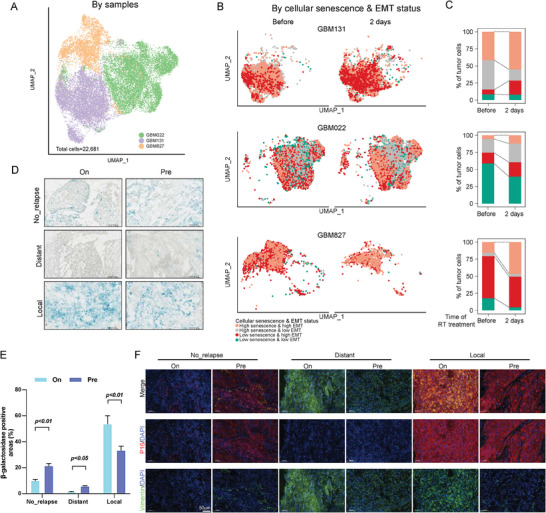
Validation of radiation‐induced changes in cellular senescence and EMT. A) A uniform manifold approximation and projection (UMAP) plot of patient‐derived glioblastoma (GBM) cells. Each dot corresponds to a single cell, color‐coded by patient ID. B) UMAP plots showing the distribution of malignant cells before and after two days of radiation in three patients. The dots are colored according to the expression levels of cellular senescence and EMT signatures. C) A histogram showing the number of malignant cells categorized by cellular senescence and EMT status for each patient. D) Representative SA‐β‐Gal activity assay images of LACC specimens in different groups. SA‐β‐Gal positive cells were stained blue. Scale bar = 100 µm. E) The percentage of SA‐β‐Gal positive cells was quantified in randomly selected fields using ImageJ software. The results are presented as mean ± standard error of the mean (s.e.m.). Statistical significance was determined using an unpaired Student's *t*‐test. F) Representative multiplexed immunofluorescence images of LACC specimens in different groups. Red: p16 (senescence marker); green: vimentin (EMT marker); blue: DAPI (nuclear stain). Scale bar = 50 µm.

To validate findings from our snRNA‐seq analysis, we analyzed scRNA‐seq data from patient‐derived glioblastoma (GBM) cells^[^ ^14^ ^]^ (**Figure** **3**A). GBM tumor tissues were cut into pieces ≈0.5–1 mm in diameter and cultured in an incubator. The patient‐derived GBM cells were exposed to 10 Gy of in vitro irradiation. Cells were collected on day 0 or day 2, digested into single‐cell suspension, and profiled using the 10× Genomics single‐cell platform.^[^ ^14^ ^]^ Consistent with our initial results, we observed an increased proportion of malignant cells exhibiting high senescence & high EMT and low senescence & high EMT statuses after radiotherapy (Figure 3B,C). To substantiate these results using non‐sequencing methods, we performed senescence‐associated β‐galactosidase (SA‐β‐gal) staining on pre‐ and on‐treatment LACC tumor samples (Figure 3D). In line with our analysis of cellular senescence gene expression, we observed SA‐β‐gal staining signals in all the LACC tumors examined. Notably, positive staining areas of SA‐β‐gal signals were increased in patients with local tumor recurrences while decreasing in those with distant ones (Figure 3E). Furthermore, multiplex immunofluorescence (mIF) staining using the senescence and EMT markers, p16 and vimentin, respectively, demonstrated that most cells in the distant recurrence group exhibited a low senescence & high EMT status. However, the cells in the local recurrence group showed a high senescence & high EMT status (Figure 3F). Collectively, these results, obtained through multiple approaches, confirmed changes in senescence and EMT expression in the tumor samples collected during treatment, highlighting that different alterations were observed across the various patterns of disease relapse in response to radiotherapy.

### 2.4 The Expression of Inflammatory and Cellular Senescence‐Related Genes in Fibroblasts was Increased in Patients who Experienced either Local or Distant Disease Relapses

**Figure 4:**
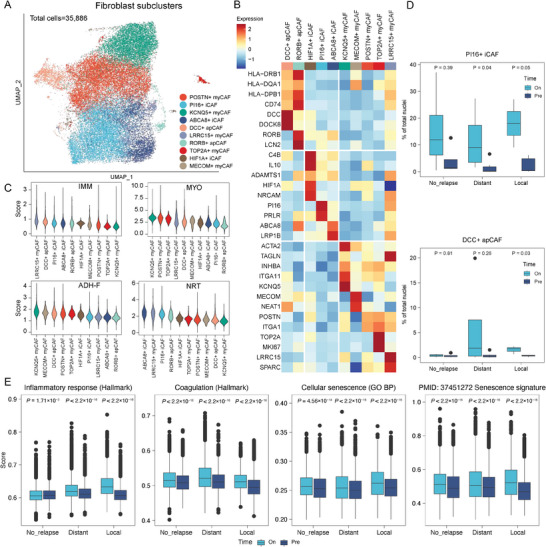
Ubiquitously upregulated expression of cellular senescence signatures in fibroblasts after radiotherapy, independent of disease relapse status. A) A uniform manifold approximation and projection (UMAP) plot of fibroblasts from all the tumor samples, showing 10 subclusters. Each dot represents a single nucleus, color‐coded by fibroblast subcluster. B) A heatmap showing the expression of cell type marker genes for each subcluster (the color legend is shared with panel A). C) Distribution of the signature scores for the CAF programs: immunomodulatory (IMM), myofibroblastic progenitor (MYO), adhesive fibroblast (ADH‐F), and neurotropic (NRT) within each fibroblast subcluster. D) Boxplots showing the proportion of fibroblast subclusters between pre‐ and on‐treatment tumors in all the different disease relapse statuses. The center line indicates the median value, with the lower and upper hinges representing the 25th and 75th percentiles, respectively, and the whiskers denoting the 1.5 interquartile range. The *P*‐values were calculated by a two‐sided Wilcoxon rank‐sum test. E) Boxplots showing the expression levels of fibroblasts in pre‐ and on‐treatment tumors in all the different disease relapse statuses. The center line indicates the median value, with the lower and upper hinges representing the 25th and 75th percentiles, respectively, and the whiskers denoting the 1.5 interquartile range. The *P*‐values were calculated by a two‐sided Wilcoxon rank‐sum test.

Previously, we dissected the radiosensitivity‐associated molecular characteristics of cancer‐associated fibroblasts (CAFs) in LACC.^[^ ^13b^ ^]^ Here, we aimed to investigate the dynamics of CAFs following radiotherapy and their potential relationship with disease relapse patterns. We performed sub‐clustering analysis and identified 10 clusters of CAFs, including 2, 3, and 5 clusters for antigen‐presenting CAF (apCAF), inflammatory CAF (iCAF), and myofibroblastic CAF (myCAF), respectively, based on established markers^[^ ^20^ ^]^ and signatures^[^ ^21^ ^]^ (**Figure** **4**A–C). In the on‐treatment samples from patients with distant tumor recurrence, we observed a higher proportion of peptidase inhibitor 16‐positive iCAF (PI16+ iCAF) cells when compared to pre‐treatment samples. Additionally, there was an increased proportion of DCC Netrin 1 Receptor‐positive apCAF (DCC+ apCAF) in the local recurrence group after radiation (Figure 4D). However, the proportions of other subclusters did not change significantly following radiotherapy (Figure S5A, Supporting Information).

We further explored whether the cellular senescence and CAF function‐related transcriptional states were associated with disease relapse patterns. We found that the expression levels of pathways related to cellular senescence, coagulation, and other types of inflammation were consistently higher in CAFs from both local and distant recurrence groups in the on‐treatment samples (Figure 4E; Figure S5B, Supporting Information). The expression of cellular senescence pathways was slightly higher in on‐treatment tumors from the local recurrence group when compared to those from the distant recurrence group (Figure 4E). Collectively, the CAFs exhibited increased expression of cellular senescence pathways following radiotherapy in all the disease‐relapse pattern groups, indicating significant alterations after radiation and weak correlations with disease relapse patterns.

### 2.5 Cell–Cell Interaction Analysis in Tumors from Patients with Different Disease Relapse Patterns Highlighted the Role of the IL1 Pathway

**Figure 5:**
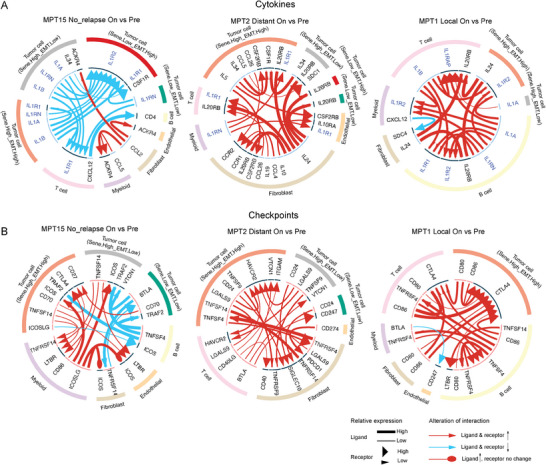
The enriched ligand–receptor cell–cell interactions across major cell types in all the different disease relapse groups. A, B) Representative circos plots showing details of the top 20 differentially regulated cytokines (A) and checkpoints (B) ligand–receptor pairs between on‐ and pre‐treatment tumors in the indicated patient. The lines are colored according to their alterations. The size of the lines indicates the relative expression of ligands. The size of the arrows indicates the relative expression of receptors.

To investigate the interactions between malignant and other cell types within the TME, we employed iTALK,^[^ ^22^ ^]^ utilizing its built‐in ligand‐receptor pair database. In patients who did not relapse, we observed a decrease in cytokine ligand‐receptors interactions between the malignant and other cell types in the on‐treatment tumors when compared to pre‐treatment ones (**Figure** **5**A; Figure S6, Supporting Information). In contrast, our analysis revealed a consistent increase in the expression of interleukin 1 (IL1)‐related ligand‐receptor pairs in malignant cells from patients with distant tumor recurrence. Patients with local tumor recurrence exhibited a broader range of cytokine‐related cell–cell interactions between the malignant and other cells in on‐treatment tumor samples when compared to pre‐treatment ones (Figure 5A; Figures S7, S8, Supporting Information). Additionally, we identified significant changes in cellular interactions involving checkpoint genes, such as PDCD1 and CD274, which were notably upregulated in on‐treatment samples when compared to the corresponding pre‐treatment samples from both the local and distant groups, but not in those from the no‐relapse group (Figure 5B; Figures S6–S8, Supporting Information). These findings indicated the presence of shared characteristics of cytokine‐ and checkpoint‐related cell–cell interactions between malignant cells and other TME component cells in both disease relapse groups.

### 2.6 Anti‐IL1R1 as a Promising Strategy for Ameliorating Radiotherapy Outcomes in LACC Patients

**Figure 6:**
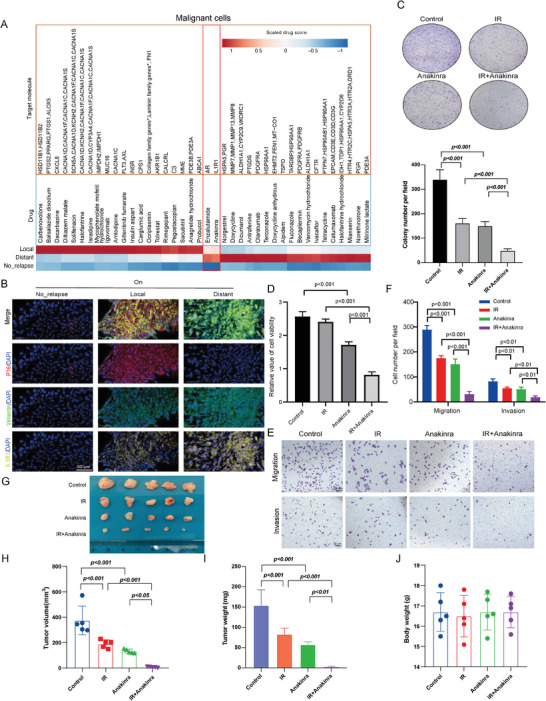
A combination of anti‐IL1R1 and radiotherapy exhibits an enhanced cancer cell‐killing effect. A) A heatmap of the significantly elevated drug scores in malignant cells from patients with local and distant tumor recurrences compared to those without disease relapse (two‐sided Wilcoxon test, *P*‐value < 0.05). The top 20 drugs for each group are illustrated based on fold changes. The heatmap colors represent z‐score scaled values for each drug. Targets with higher scores in both local and distant groups are highlighted in a red box. B) Representative multiplexed immunofluorescence images of LACC on‐treatment specimens in different disease relapse groups. Red: p16 (senescence marker); green: vimentin (EMT marker); yellow: IL1R1; blue: DAPI (nuclear stain). Scale bar = 50 µm. C) Crystal violet staining was performed to assess cell colonies in all the different groups (n = 3). Cells were treated with anakinra before exposure to 6 Gy of IR. The cells were fixed and stained two weeks after IR treatment. The results are presented as mean ± s.e.m. Statistical significance was determined using one‐way ANOVA followed by Tukey's multiple comparisons test. D) Cell viability was assessed using the CCK‐8 assay in the specified treatment groups (one‐way ANOVA followed by Tukey's multiple comparisons test, mean ± s.e.m). Cells were treated with anakinra (10 µg mL^−1^), either alone or in combination with ionizing radiation (6 Gy) as indicated. Anakinra was reapplied on the second day. The assays were performed 48 h after IR treatment. E) Representative images from the trans‐well assays showing the effects of anakinra on the migratory and invasive abilities of irradiated SiHa cells; Scale bar = 20 µm. F) Quantitative analysis of migrated or invaded cells per field across treatment groups (one‐way ANOVA followed by Tukey's multiple comparisons test, mean ± s.e.m). G) Representative images of subcutaneous tumor formation in the specified treatment groups. H, I) Quantification of tumor volume (H) and tumor weight (I) for subcutaneous tumor formations. The results are shown as the mean ± s.e.m. Statistical significance was determined using one‐way ANOVA followed by Tukey's multiple comparisons test. J) Quantification of body weight of mice for toxicity evaluation. The results are shown as the mean ± s.e.m.

Next, we sought to leverage our single‐nucleus resolution transcriptomic data to identify potential intervention strategies for tumor recurrence after radiotherapy. We employed the Python package drug2cell^[^ ^23^ ^]^ to evaluate drug‐target expression by integrating drug‐target interactions from the ChEMBL database with single‐cell data at the individual cell level. Analysis of malignant cells from patients with local and distant tumor recurrence after radiotherapy, as characterized predominantly by high senescence & high EMT and low senescence & high EMT statuses, respectively, revealed distinct therapeutic targets. Notably, the collagen and matrix metallopeptidase families of genes were identified in the local and distant recurrence groups, respectively (**Figure** **6**A). Similarly, we identified different targets associated with each relapse pattern in the CAFs, such as hypoxia inducible factor 1 subunit alpha (HIF1A), spleen associated tyrosine kinase (SYK) and interleukin 4 receptor (IL4R) as targets in the local, distant and both groups, respectively (Figure S9, Supporting Information).

Consistent with our cell–cell interaction analysis, we found that IL1R1 might be a potential drug target in malignant cells from both the local and distant recurrence groups (Figure 6A). Additionally, we performed mIF on tumor tissues from LACC patients, using antibodies against IL1R1, p16, and vimentin. This analysis showed stronger staining signals of IL1R1, along with corresponding changes in p16 and vimentin, in patients with local or distant disease relapse (Figure 6B). Previous studies have implicated the role of the IL‐1 signaling pathway in mediating cellular senescence in irradiated inflammatory fibroblasts in rectal cancer.^[^ ^24^ ^]^ Moreover, IL‐1β, a key component of the IL‐1 signaling pathway, has been shown to promote the EMT.^[^ ^25^ ^]^ Therefore, we tested the effect of the IL1R1 inhibitor, anakinra, in killing malignant cells with or without radiation both in vitro and in vivo. To link EMT and senescence pathway changes to disease relapse, we conducted colony formation, CCK‐8, and trans‐well assays to assess the effects of anakinra on cell proliferation, migration, and invasion of irradiated SiHa cells. Anakinra significantly reduced their cell proliferation abilities (Figure 6C,D), aligning with its inhibitory effects on cell migration and invasion abilities (Figure 6E,F). Furthermore, a subcutaneous tumor‐xenograft BALB/c nude mouse model demonstrated superior in vivo irradiated tumor‐killing ability with a combination of anakinra administration (Figure 6G–I). The body weights of mice across different treatment groups did not significantly decrease (Figure 6J) and no pathological damages were detected in the major organs of these mice (Figure S10, Supporting Information), suggesting minimal toxicities of these treatments. In summary, the ability of anakinra to kill irradiated tumor cells in both in vitro and in vivo models suggested its potential as a promising therapeutic agent for the treatment of LACC patients with either local or distant tumor recurrences after radiotherapy.

### 2.7 Impact of Anti‐IL1R1 on Cellular Senescence and EMT Pathways

**Figure 7:**
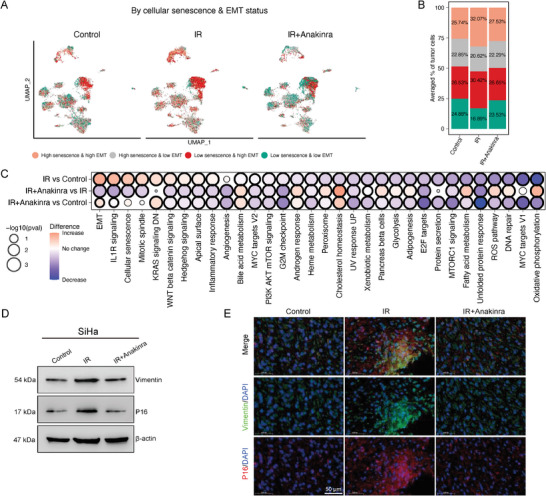
IL1R1 inhibition reduces cellular senescence and EMT pathways in irradiated tumor cells. A) UMAP plots showing the distribution of SiHa cells in the different treatment groups. Cells are colored according to their expression levels of cellular senescence and EMT signatures, as determined by scRNA‐seq results. B) Histogram depicting the proportions of cells categorized based on their cellular senescence and EMT status across treatment groups. C) Differential expression of EMT, cellular senescence, IL1R pathway, and hallmark gene sets across each treatment group. Dot size represents the significance (*P*‐values), and dot color indicates the magnitude of expression change between groups. The *P*‐values were calculated by a two‐sided Wilcoxon rank‐sum test. D) Immunoblot analysis showing the expression levels of p16 and vimentin expression in SiHa cells from different treatment groups. E) Representative multiplexed immunofluorescence images of tumor xenograft of BALB/c nude mice treated by different therapies. Red: p16 (senescence marker); green: vimentin (EMT marker); blue: DAPI (nuclear stain). IR: ionizing radiation. Scale bar = 50 µm.

To further elucidate the mechanistic role of IL1R1 inhibition, we performed scRNA‐seq analysis on SiHa cells across different treatment groups, successfully identifying 33944 cells and 10 subclusters (Figure S11A–C, Supporting Information). We scored each SiHa cell for the expression of EMT modules,^[^ ^15^ ^]^ IL1R pathway, cellular senescence pathway, and hallmark gene sets from MsigDB.^[^ ^17^ ^]^ Consistently, we observed increased proportions of cells with high senescence & high EMT and low senescence & high EMT statuses after IR (**Figure** **7**A,B). Differential expression analysis identified EMT, cellular senescence, and IL1R pathways among the top 5 upregulated pathways after irradiation (Figure 7C). Treatment with anakinra effectively reduced IL1R pathway expression, confirming its therapeutic potential. In addition, the expression of EMT and senescence pathways was also significantly decreased when IR+anakinra was administered (Figure 7C; Figure S11D–F, Supporting Information). These findings were further supported by immunoblot analysis, which demonstrated elevated levels of p16 and vimentin in irradiated tumor cells that were markedly reduced after anakinra treatment (Figure 7D). Moreover, mIF analysis of subcutaneous tumor xenografts in BALB/c nude mice revealed increased staining of p16 and vimentin in the IR group, which was reversed with IR+anakinra treatment (Figure 7E). Taken together, these results suggest that irradiation enhances the expression of cellular senescence and EMT‐related markers, and IL1R1 inhibition via anakinra could mitigate these effects.

## 3 Discussion

The relapse rate of LACC patients after radiotherapy ranges from 20% to 40%.^[^ ^26^ ^]^ However, the underlying dynamics and phenotypic trajectories of the TME that contribute to tumor recurrence remain poorly understood. In this study, we conducted a comprehensive snRNA‐seq analysis of paired pre‐ and on‐radiotherapy LACC tumor samples, encompassing multiple disease relapse pattern subgroups, to better characterize the complex TME landscape following radiation treatment. Our analysis revealed several important findings. The expression levels of the cellular senescence pathway in malignant cells showed varying trends among patients with different disease relapse patterns, displaying opposite directions in those with local vs distant relapse following radiotherapy. In contrast, the expression of the EMT module was consistently elevated in malignant cells from on‐treatment tumors compared to pre‐treatment ones in patients with any type of disease relapse. These findings suggest that the combined EMT and cellular senescence profiles of malignant cells may play pivotal roles in disease relapse after radiotherapy. Notably, the cellular senescence status of CAF ubiquitously increased following radiotherapy, regardless of whether patients experienced relapse. These findings provide insight into the associations between the cellular senescence of various cell types within TME and the prognosis of LACC patients. Collectively, our study delineates the cellular senescence landscapes of both CAF and malignant cells following radiotherapy and deepens the understanding of the interplay between senescence, EMT, and distinct disease relapse patterns. Additionally, we validated whether anakinra, an IL1R1 inhibitor, could be a potential therapeutic agent to improve the outcomes in LACC patients receiving radiotherapy. This study highlights the significance of targeting the EMT as well as cellular senescence‐associated pathways in malignant cells as a potential strategy to reduce the risk of relapse in LACC patients undergoing radiotherapy.

Several recent analyses have reported the association of cellular senescence with therapy efficacy and tumorigenesis. For instance, one study with a mouse model reported that irradiated SA‐β‐gal+ GBM cells undergo clonal expansion and promote tumor regrowth after radiation therapy.^[^ ^14^ ^]^ Similarly, macrophages expressing senescence‐associated gene markers have been identified in human pre‐malignant lung tumors.^[^ ^27^ ^]^ In rectal cancer, another study with a mouse model suggested that irradiation of inflammatory fibroblasts induced cellular senescence through an IL‐1‐dependent signaling pathway.^[^ ^24^ ^]^ In our study on LACC, we found that the patients who experienced disease relapse exhibited increased expression of cellular senescence pathways in their malignant cells, together with enhanced cell–cell interactions involving the IL‐1 signaling pathway ligand‐receptor pairs. In addition, significant changes in cellular interactions involving checkpoint genes suggest potential benefits of combining radiotherapy and immune checkpoint inhibitors, which are currently undergoing clinical trials. Furthermore, we demonstrated that the IL1R1 inhibitor, anakinra, synergized with IR to effectively kill cervical cancer cells. However, due to the lack of mouse models that could realistically represent different disease relapse patterns of LACC, we were unable to explore the precise mechanism of how the IL‐1 pathway mediates the senescence of malignant cells during tumor recurrence.

EMT is a crucial process in cancer progression. It transforms epithelial cells into a more aggressive state, enhancing their ability to migrate and invade tissues and resist cancer therapies. This transformation contributes significantly to tumor recurrence and metastasis. Recent studies have indicated that EMT is closely associated with malignant cell lineage plasticity during anti‐cancer treatment, facilitating the emergence of drug resistance.^[^ ^28^ ^]^ Various factors in the TME, such as growth factors, hypoxia, and inflammatory cytokines can trigger EMT. Promotion of the EMT activates anti‐apoptotic pathways and contributes to the formation of cancer stem cells, giving tumor cells a survival advantage, particularly under stressful conditions such as radiation therapy. Coincidently, radiotherapy has long been recognized for its ability to enhance the secretion of various chemokines, cytokines, and extracellular matrix molecules by malignant cells, leading to the remodeling of the TME.^[^ ^29^ ^]^ Among these factors, IL1B has been demonstrated as a crucial element in inducing the EMT process of cancer cells. For example, in hepatocellular carcinoma, IL1B from tumor‐activated monocytes promoted cancer cell autophagy in the invading edge regions, further driving EMT of cancer cells and tumor metastasis.^[^ ^25a^ ^]^ In line with this, we observed consistently increased expression of EMT modules in malignant cells from on‐radiation tumors of patients with worse outcomes. However, further studies are necessary to explore the specific pathways associated with EMT that influence radio‐sensitivity.

There are several limitations to this study. The technical constraints of snRNA‐seq limited our ability to obtain relapse‐related information from the perspective of immune cells. Although scRNA‐seq could capture a more comprehensive immune cell profile, it requires fresh tumor samples, making long‐term studies (over a period of at least 2 years) to observe disease relapse impractical. In addition, if we used dissociation‐free sample preparation methods, this could have potentially yielded relatively accurate molecular profiles of cells. Nonetheless, despite these challenges, our study of a limited‐sized snRNA‐seq cohort was the largest single‐cell resolution cohort that focused on disease relapse in LACC. We also used a cancer cell line and mouse model to confirm and consolidate our observations. Our future investigations will include a larger cohort of patients with diverse disease relapse patterns in order to explore the recurrent related lineage plasticity of senescent malignant cells upon radiation in a more comprehensive manner.

In summary, we have elucidated the malignant cell state transitions that occur during the adaption of LACC patients who undergo radiation therapy. These differed substantially among patients with different sites of tumor recurrence. Our findings offer a step forward in understanding the mechanisms underlying different disease relapse patterns after radiotherapy, which may be critical for improving the treatment outcomes of LACC patients.

## 4 Experimental Section

### Patient Populations

Between Aug 27, 2019, and Oct 29, 2021, cervical cancer tissue samples were collected from 17 patients with LACC. Sixteen patients underwent radical radiotherapy and one received chemotherapy after external beam radiation. The treatment consisted of a 45 Gy external beam radiotherapy (EBRT) given at 5 fractions per week, followed by image‐guided brachytherapy which consisted of 30–35 Gy in 5–6 fractions. 11 of the patients received concurrent chemotherapy with cisplatin, and 11 patients also received adjuvant chemotherapy post radiotherapy. The characteristics of individual patients are given in Table S1 (Supporting Information). The cohort consisted of 28 samples, including 22 matched pre‐ and post‐radiotherapy tumor tissues from 11 patients, which were used for single‐nucleus analysis. At the data cutoff (Dec 31, 2023), all the patients were categorized into three groups based on disease relapse patterns: loco‐regional tumor recurrences (Local, n = 5), distant tumor recurrences (Distant, n = 7) and no tumor recurrences (No relapse, n = 5). Loco‐regional tumor recurrence (local recurrence) was defined as tumor recurrence within the pelvic region, including areas such as the uterus, cervical, vaginal, para‐cervical tissue, pelvic wall, or pelvic lymph node, as well as the paraaortic lymph node area. Distant tumor recurrence was defined as tumor recurrence in other regions, such as distant organs (e.g., lung, liver, brain, bone) or lymph nodes located above the renal veins (Figure S1, Supporting Information). The research was conducted with approval from the Ethics Committee of Renji Hospital, Shanghai Jiao Tong University School of Medicine (KY2021‐268‐B) and all the patients provided written consent for the use of their tissue samples in the study.

### Single‐Nucleus and Single‐Cell RNA Sequencing

Nuclei were extracted from frozen cervical cancer tumor samples using standard protocols to maintain their integrity. The samples were mechanically dissociated with the Shbio Cell Nuclear Isolation Kit (Shbio, 52009–10, Shanghai, China) and then these were filtered through 40 µm cell strainers to remove debris and aggregates. Nucleus quality was assessed using a hemocytometer and microscopy to ensure a high yield of intact nuclei. Subsequently, snRNA‐seq was performed using the Chromium Single Cell 3ʹ Reagent Kit v3 (10× Genomics, 1000121, USA) following the manufacturer's instructions. The libraries were sent for sequencing on an Illumina NovaSeq 6000 platform and this was performed by the Shanghai Biotechnology Corporation. Single‐cell suspension of SiHa cells were prepared by using the DNBelab C‐series high‐throughput single‐cell RNA library preparation kit (Mgitech, 940‐000051‐00, Qingdao, China). Cell suspensions were processed through the DNBelab C4 system to create water‐in‐oil droplets, followed by demulsification. The resulting products underwent reverse transcription, cDNA and oligo product amplification, and library construction. Sequencing and data analysis were conducted using the MGISEQ‐2000RS sequencing platform.

### snRNA‐Seq/scRNA‐Seq Data Preprocessing

The CellRanger pipeline (version 7.1.0, 10x Genomics) was utilized to process the raw sequencing data with default parameters for de‐multiplexing, barcode processing, and gene counting (GRCh38 as reference). Quality control was performed using the Seurat package (version 4.1.0) in R. To ensure data quality, nuclei or cells were excluded with fewer than 200 detected genes and those where more than 10% of reads mapped to mitochondrial genes from subsequent analysis. The remaining expression data were then merged and batch‐corrected by Harmony (v1.2.0).

### Dimensionality Reduction, Clustering, and Annotation

Principal component analysis (PCA) was conducted on the normalized and batch‐corrected data to identify the primary sources of variations. The top significant principal components were selected for further analysis. Uniform manifold approximation and projection (UMAP) was then used to reduce dimensions and facilitate visualization, thereby effectively representing the high‐dimensional data in two dimensions. Nuclei and cells were clustered using the Louvain algorithm, which grouped them based on transcriptional similarities. Cell types were annotated by referencing known marker genes and refining these annotations with canonical markers and biological insights. The marker genes used for cell type identification included fibroblasts (COL1A1 and COL1A2), pericytes (KCNJ8, ABCC9, CSPG4, and RGS5), endothelial cells (RAMP2, FLT1, CLDN5, and PECAM1), epithelial cells (EPCAM, KRT5, KRT14, and CDH1), T cells (CD3G, CD3E, CD4, CD8A, and CD8B), myeloid cells (FCGR3A, CD14, MARCO, and CD68), B cells (CD19, CD79A and MS4A1), as well as mast cells (GATA2, MS4A2 and KIT). Additionally, pathway scores for each cell was calculated using the ssGSVA function from the GSVA software package.

### Copy Number Variation Analysis using inferCNV

Copy number variations (CNVs) were assessed using the inferCNV R package (version 1.2.1; https://github.com/broadinstitute/inferCNV) to distinguish between malignant and non‐malignant epithelial cells. CNVs were inferred by comparing read‐depth variations across genomic positions between tumor and stromal cells. Malignant cells were differentiated from normal cells by integrating information from various sources, including cell cluster distribution, marker gene expression, and inferred large‐scale CNVs.

### Cell–Cell Interaction Analysis

Cell‐to‐cell communication in LACC tumors was analyzed using the iTALK software package in R.^[^ ^22^ ^]^ This tool evaluates interaction networks between different cell types in the tumor environment based on known receptor‐ligand pairs. Gene expression profiles from annotated cell types were used as the input data. The *P*‐values were used to determine whether the results were statistically significant.

### CCK‐8 Assay

Cell Counting Kit‐8 (CCK‐8) assay was used for assessing cell viability. A total of 5000 cells per well were suspended in 100 µL of medium and plated in 96‐well plates. After overnight incubation, cells were adhered to the plate, and anakinra (10 µg Ml^−1^) was applied, either alone or in combination with ionizing radiation (6 Gy), as specified for each group. Anakinra treatment was repeated on the second day. After 48 h of treatment, 10 µL of CCK‐8 reagent was added to each well, and the plates were incubated for 1 h at 37 °C. Then a microplate reader was used to measure the absorbance at 450 nm.

### Colony Formation Assay

To evaluate the colony‐forming ability of cancer cells, a colony‐formation assay was conducted. Cells were seeded into 6‐well plates at a density of 1000 cells per well and treated according to the CCK‐8 methodology for the specified groups. Then cells were cultured under suitable conditions for 2 weeks to allow colony development. The colonies were fixed with 4% paraformaldehyde for 10 min, stained with 0.5% crystal violet for another 10 min, and then washed with distilled water. Digital images were captured using a digital camera, and colonies, which were defined as groups of more than 50 cells, were counted using ImageJ software (Fiji, version 2.12.0).

### Western Blot Analysis

Western blot analysis was performed following standard protocols. Briefly, protein samples (50 µg) from different treatment groups were separated by SDS‐PAGE and transferred to polyvinylidene difluoride (PVDF) membranes. The membranes were blocked with 5% non‐fat milk and then incubated overnight at 4 °C with the following primary antibodies: p16 (1:500, Abcam, ab108349), vimentin (1:500, Abcam, ab92547), and β‐actin (1:2000, Sigma, A5441). After washing with PBS‐T, the membranes were incubated with the corresponding secondary antibodies (1:5000) for 1 h at room temperature. After additional washes with PBS‐T, an enhanced chemiluminescence (ECL) solution (Thermo Scientific) was applied, and protein bands were visualized using the Tanon 5200 system (Tanon, Shanghai, China).

### Trans‐Well Assay

Cell migration and invasion were evaluated using 24‐well trans‐well plates (BD Biosciences, CA, USA). Cells were treated according to the CCK‐8 methodology for the specified groups. For the migration assay, 4 × 10^4^ SiHa cells suspended in 0.2 mL DMEM were added to the upper chamber. For the invasion assay, 6 × 10^4^ cells in 0.2 mL DMEM were seeded in the upper chamber, which was pre‐coated with Matrigel (BD Biosciences, CA, USA). The lower chamber contained 0.8 mL DMEM supplemented with 10% fetal bovine serum. After 24 h of incubation at 37 °C, non‐migrated and non‐invaded cells were removed from the upper chamber using a cotton swab. The remaining cells were fixed and stained with 1% crystal violet in methanol for 10 min, and visualized with a light microscope (Olympus, Japan).

### Subcutaneous Tumor Formation in Mice and Histological Analysis

All animal procedures were approved by the Ethics Committee at Renji Hospital, Shanghai Jiao Tong University School of Medicine. Subcutaneous tumor formation was performed in mice to assess the in vivo tumorigenic potential. Female BALB/c nude mice, aged 4–6 weeks, were injected subcutaneously in the right flank with 1 × 10^6^ cancer cells suspended in Matrigel. After 7 days of tumor cell implantation, mice were randomized into four groups of five mice each. One group of mice was treated with saline control, and the other three groups were treated with ionizing radiation (12 Gy), anakinra (intraperitoneal injection 200 µL of anakinra solution from MedChemExpress, HY‐108841, at a dosage of 1.5 mg kg^−1^ day^−1^ for 5 consecutive days), or a combination of anakinra and ionizing radiation, respectively. Ionizing radiation (12 Gy) was administered on day 9. The mice were humanely euthanized on day 21, and tumors were surgically removed for analysis. Tumor volume and weight were measured and these were subsequently analyzed. To evaluate potential toxicity, major organs (heart, liver, spleen, lung, and kidney) were collected from the BALB/c nude mice for histological analysis (H & E staining). Following standard protocols, the tissue samples were fixed, embedded in paraffin, and sectioned into 5 µm slices. After staining with hematoxylin and eosin, the sections were examined and imaged using a light microscope (Olympus, Japan).

### Multiple Immunofluorescence Staining

Multiple immunofluorescence staining on cancer tissue sections was conducted to show the expression of the senescence and EMT markers, p16 and vimentin, respectively. The tissue sections were deparaffinized, rehydrated, and underwent antigen retrieval using citrate buffer (pH 6.0) at a high temperature. To prevent non‐specific binding, the sections were treated with 5% bovine serum albumin for 1 h, followed by an overnight incubation at 4 °C with primary antibodies (p16, 1:100, Abcam, ab108349; vimentin, 1:100, Abcam, ab92547; IL1R1, 1:100, Abcam, ab106278) targeting the proteins of interest. The next day, the sections were washed and then these were incubated with fluorophore‐conjugated secondary antibodies for 1 h at 25 °C. Nuclei were counterstained with DAPI, and the slides were mounted using an anti‐fade medium before capturing the fluorescence signals.

### Senescence‐Associated β‐Galactosidase (SA‐β‐Gal) Activity Assay

The Senescence β‐Galactosidase Staining Kit (C0602, Beyotime, Shanghai, China) was used to evaluate cellular senescence in cancer tissue samples. Tissue sections (5 µm thick) were prepared from paraffin blocks and mounted on positively charged glass slides. After de‐paraffinization and rehydration, the sections were fixed for 30 min and washed with PBS. They were then incubated at 37 °C in a staining solution for SA‐β‐Gal. The reaction proceeded for 24 h. The stained sections were then examined under a bright‐field microscope. The percentage of SA‐β‐Gal positive cells (blue‐stained) was analyzed using ImageJ software (Fiji, 2.12.0).

### Statistical Analysis

All statistical analyses were conducted using R (version 4.3.0) or GraphPad Prism (version 8.0.2). For snRNA‐seq and scRNA‐seq data, Unique Molecular Identifier (UMI) counts were transformed and normalized using the “NormalizeData” function in the Seurat package (version 4.1.0), with the normalization method set to “logNormalize” and the scale factor set to 10000. Nuclei or cells with fewer than 200 detected genes and those with over 10% of reads mapping to mitochondrial genes were excluded from subsequent analysis. Results are presented as the mean ± standard error of the mean (s.e.m.) unless otherwise indicated in figure legends. Sample sizes for each statistical analysis are provided in the corresponding figure or figure legends. For comparisons between different disease relapse groups in scRNA‐seq and snRNA‐seq, the two‐sided Wilcoxon rank‐sum test was employed. For comparisons of SA‐β‐Gal positive cells in tumor tissues before and during radiotherapy, an unpaired Student's *t*‐test was used. For in vivo and in vitro experiments, one‐way ANOVA followed by Tukey's multiple comparisons test was performed. A *P*‐value of 0.05 or less was considered statistically significant.

### Data Availability

The data of snRNA‐seq of LACC patients are available in the Genome Sequence Archive (HRA008063, accession numbers were listed in Table S2, Supporting Information). The data for scRNA‐seq of SiHa cells are available in the Genome Sequence Archive (HRA009490). However, access to these data is restricted as they were used under license for this study. Please contact the authors, who can provide access with permission from the Genome Sequence Archive, to obtain the original data. The single‐cell raw expression matrices of the GBM that support the findings of this study are available on the Gene Expression Omnibus (GSE162931).

## Conflict of Interest

The authors declare no conflict of interest.

## Author Contributions

L.Z. and J.M. contributed equally to this work. Y.J., H.C., and L.Z. conceived of the idea for the project. Y.J. and J.Z. analyzed the data. L.Z., M.H., J.C., and J.M. performed experiments. and B.H., J.Z., D.Z., X.M., Y.B., J.T., and H.C. provided samples. L.Z., J.M., and Y.J. wrote the manuscript. All authors provided feedback and edited the manuscript.

## Supporting information



Supporting Information

## Data Availability

The data that support the findings of this study are openly available in [Genome Sequence Archive] at [https://www.ngdc.cncb.ac.cn/gsa/], reference number [HRA008063 and HRA009490]

## References

[1] R. L. Siegel , K. D. Miller , H. E. Fuchs , A. Jemal , CA Cancer J. Clin. 2022, 72, 7. 35020204 10.3322/caac.21708

[2] C. Gennigens , M. De Cuypere , J. Hermesse , F. Kridelka , G. Jerusalem , Expert Rev. Anticancer Ther. 2021, 21, 657. 33472018 10.1080/14737140.2021.1879646

[3] P. Sidaway , Nat. Rev. Clin. Oncol. 2024, 21, 402. 10.1038/s41571-024-00889-9 38565936

[4] a) T. H. Kim , M. H. Kim , B. J. Kim , S. I. Park , S. Y. Ryu , C. K. Cho , Int. J. Radiat. Oncol. Biol. Phys. 2017, 98, 1124; 28721896 10.1016/j.ijrobp.2017.03.029

[5] a) E. E. Harris , W. T. Hwang , F. Seyednejad , L. J. Solin , Cancer. 2003, 98, 2144; 14601083 10.1002/cncr.11767

[6] a) L. Bejarano , M. J. C. Jordao , J. A. Joyce , Cancer Discov. 2021, 11, 933; 33811125 10.1158/2159-8290.CD-20-1808

[7] Z. Yuan , Y. Li , S. Zhang , X. Wang , H. Dou , X. Yu , Z. Zhang , S. Yang , M. Xiao , Mol. Cancer. 2023, 22, 48. 36906534 10.1186/s12943-023-01744-8 PMC10007858

[8] a) C. Fouillade , S. Curras‐Alonso , L. Giuranno , E. Quelennec , S. Heinrich , S. Bonnet‐Boissinot , A. Beddok , S. Leboucher , H. U. Karakurt , M. Bohec , S. Baulande , M. Vooijs , P. Verrelle , M. Dutreix , A. Londono‐Vallejo , V. Favaudon , Clin. Cancer Res. 2020, 26, 1497; 31796518 10.1158/1078-0432.CCR-19-1440

[9] a) E. Mavrogonatou , H. Pratsinis , D. Kletsas , Semin. Cancer Biol. 2020, 62, 182; 31260734 10.1016/j.semcancer.2019.06.018

[10] a) C. Carl , A. Flindt , J. Hartmann , M. Dahlke , D. Rades , J. Dunst , H. Lehnert , F. Gieseler , H. Ungefroren , Cell. Mol. Life Sci. 2016, 73, 427; 26238393 10.1007/s00018-015-2003-2 PMC11108547

[11] S. Zhou , M. Zhang , C. Zhou , W. Wang , H. Yang , W. Ye , Crit. Rev. Oncol. Hematol. 2020, 150, 102961. 32361589 10.1016/j.critrevonc.2020.102961

[12] J. Verstappe , G. Berx , Semin. Cancer Biol. 2023, 90, 15. 36773819 10.1016/j.semcancer.2023.02.001

[13] a) Cancer Genome Atlas Research Network , Albert Einstein College of Medicine , Analytical Biological Services , Barretos Cancer Hospital , Baylor College of Medicine , Beckman Research Institute of City of Hope , Buck Institute for Research on Aging , Canada's Michael Smith Genome Sciences Centre , Harvard Medical School , Helen F. Graham Cancer Center & Research Institute at Christiana Care Health Services , HudsonAlpha Institute for Biotechnology , ILSbio , LLC , Indiana University School of Medicine , Institute of Human Virology , Institute for Systems Biology , International Genomics Consortium , Leidos Biomedical , Massachusetts General Hospital , McDonnell Genome Institute at Washington University , Medical College of Wisconsin , Washington University in St. Louis , Nature. 2017, 543, 378;28112728

[14] H. M. Jeon , J. Y. Kim , H. J. Cho , W. J. Lee , D. Nguyen , S. S. Kim , Y. T. Oh , H. J. Kim , C. W. Jung , G. Pinero , T. Joshi , D. Hambardzumyan , T. Sakaguchi , C. G. Hubert , T. M. McIntyre , H. A. Fine , C. L. Gladson , B. Wang , B. W. Purow , J. B. Park , M. J. Park , D. H. Nam , J. Lee , Cancer Cell. 2023, 41, 1480. 37451272 10.1016/j.ccell.2023.06.007 PMC10530238

[15] D. Barkley , R. Moncada , M. Pour , D. A. Liberman , I. Dryg , G. Werba , W. Wang , M. Baron , A. Rao , B. Xia , G. S. Franca , A. Weil , D. F. Delair , C. Hajdu , A. W. Lund , I. Osman , I. Yanai , Nat. Genet. 2022, 54, 1192. 35931863 10.1038/s41588-022-01141-9 PMC9886402

[16] D. Saul , R. L. Kosinsky , E. J. Atkinson , M. L. Doolittle , X. Zhang , N. K. LeBrasseur , R. J. Pignolo , P. D. Robbins , L. J. Niedernhofer , Y. Ikeno , D. Jurk , J. F. Passos , L. J. Hickson , A. Xue , D. G. Monroe , T. Tchkonia , J. L. Kirkland , J. N. Farr , S. Khosla , Nat. Commun. 2022, 13, 4827. 35974106 10.1038/s41467-022-32552-1 PMC9381717

[17] A. Liberzon , C. Birger , H. Thorvaldsdottir , M. Ghandi , J. P. Mesirov , P. Tamayo , Cell Syst. 2015, 1, 417. 26771021 10.1016/j.cels.2015.12.004 PMC4707969

[18] X. Qiu , Q. Mao , Y. Tang , L. Wang , R. Chawla , H. A. Pliner , C. Trapnell , Nat. Methods. 2017, 14, 979. 28825705 10.1038/nmeth.4402 PMC5764547

[19] G. S. Gulati , S. S. Sikandar , D. J. Wesche , A. Manjunath , A. Bharadwaj , M. J. Berger , F. Ilagan , A. H. Kuo , R. W. Hsieh , S. Cai , M. Zabala , F. A. Scheeren , N. A. Lobo , D. Qian , F. B. Yu , F. M. Dirbas , M. F. Clarke , A. M. Newman , Science. 2020, 367, 405. 31974247 10.1126/science.aax0249 PMC7694873

[20] D. S. Foster , M. Januszyk , D. Delitto , K. E. Yost , M. Griffin , J. Guo , N. Guardino , A. E. Delitto , M. Chinta , A. R. Burcham , A. T. Nguyen , K. E. Bauer‐Rowe , A. L. Titan , A. Salhotra , R. E. Jones , O. da Silva , H. G. Lindsay , C. E. Berry , K. Chen , D. Henn , S. Mascharak , H. E. Talbott , A. Kim , F. Nosrati , D. Sivaraj , R. C. Ransom , M. Matthews , A. Khan , D. Wagh , J. Coller , et al., Cancer Cell. 2022, 40, 1392. 36270275 10.1016/j.ccell.2022.09.015 PMC9669239

[21] W. L. Hwang , K. A. Jagadeesh , J. A. Guo , H. I. Hoffman , P. Yadollahpour , J. W. Reeves , R. Mohan , E. Drokhlyansky , N. Van Wittenberghe , O. Ashenberg , S. L. Farhi , D. Schapiro , P. Divakar , E. Miller , D. R. Zollinger , G. Eng , J. M. Schenkel , J. Su , C. Shiau , P. Yu , W. A. Freed‐Pastor , D. Abbondanza , A. Mehta , J. Gould , C. Lambden , C. B. M. Porter , A. Tsankov , D. Dionne , J. Waldman , M. S. Cuoco , et al., Nat. Genet. 2022, 54, 1178. 35902743 10.1038/s41588-022-01134-8 PMC10290535

[22] A. Sinjab , G. Han , W. Treekitkarnmongkol , K. Hara , P. M. Brennan , M. Dang , D. Hao , R. Wang , E. Dai , H. Dejima , J. Zhang , E. Bogatenkova , B. Sanchez‐Espiridion , K. Chang , D. R. Little , S. Bazzi , L. M. Tran , K. Krysan , C. Behrens , D. Y. Duose , E. R. Parra , M. G. Raso , L. M. Solis , J. Fukuoka , J. Zhang , B. Sepesi , T. Cascone , L. A. Byers , D. L. Gibbons , J. Chen , et al., Cancer Discov. 2021, 11, 2506. 33972311 10.1158/2159-8290.CD-20-1285 PMC8487926

[23] K. Kanemaru , J. Cranley , D. Muraro , A. M. A. Miranda , S. Y. Ho , A. Wilbrey‐Clark , J. Patrick Pett , K. Polanski , L. Richardson , M. Litvinukova , N. Kumasaka , Y. Qin , Z. Jablonska , C. I. Semprich , L. Mach , M. Dabrowska , N. Richoz , L. Bolt , L. Mamanova , R. Kapuge , S. N. Barnett , S. Perera , C. Talavera‐Lopez , I. Mulas , K. T. Mahbubani , L. Tuck , L. Wang , M. M. Huang , M. Prete , S. Pritchard , et al., Nature. 2023, 619, 801. 37438528 10.1038/s41586-023-06311-1 PMC10371870

[24] A. M. Nicolas , M. Pesic , E. Engel , P. K. Ziegler , M. Diefenhardt , K. B. Kennel , F. Buettner , C. Conche , V. Petrocelli , E. Elwakeel , A. Weigert , A. Zinoveva , M. Fleischmann , B. Haupl , C. Karakutuk , H. Bohnenberger , M. H. Mosa , L. Kaderali , J. Gaedcke , M. Ghadimi , F. Rodel , M. C. Arkan , T. Oellerich , C. Rodel , E. Fokas , F. R. Greten , Cancer Cell. 2022, 40, 168. 35120600 10.1016/j.ccell.2022.01.004

[25] a) D. P. Chen , W. R. Ning , X. F. Li , Y. Wei , X. M. Lao , J. C. Wang , Y. Wu , L. Zheng , Autophagy. 2018, 14, 1335; 29940792 10.1080/15548627.2018.1474994 PMC6103724

[26] F. Coutinho , M. Gokhale , C. Doran , M. Monberg , K. Yamada , L. Chen , Cancer Treat Res. Commun. 2024, 39, 100800. 38430610 10.1016/j.ctarc.2024.100800

[27] S. Haston , E. Gonzalez‐Gualda , S. Morsli , J. Ge , V. Reen , A. Calderwood , I. Moutsopoulos , L. Panousopoulos , P. Deletic , G. Carreno , R. Guiho , S. Manshaei , J. M. Gonzalez‐Meljem , H. Y. Lim , D. J. Simpson , J. Birch , H. A. Pallikonda , T. Chandra , D. Macias , G. J. Doherty , D. M. Rassl , R. C. Rintoul , M. Signore , I. Mohorianu , A. N. Akbar , J. Gil , D. Munoz‐Espin , J. P. Martinez‐Barbera , Cancer Cell. 2023, 41, 1242. 37267953 10.1016/j.ccell.2023.05.004

[28] G. S. Franca , M. Baron , B. R. King , J. P. Bossowski , A. Bjornberg , M. Pour , A. Rao , A. S. Patel , S. Misirlioglu , D. Barkley , K. H. Tang , I. Dolgalev , D. A. Liberman , G. Avital , F. Kuperwaser , M. Chiodin , D. A. Levine , T. Papagiannakopoulos , A. Marusyk , T. Lionnet , I. Yanai , Nature. 2024, 631, 876. 38987605 10.1038/s41586-024-07690-9 PMC11925205

[29] L. Galluzzi , M. J. Aryankalayil , C. N. Coleman , S. C. Formenti , Nat. Rev. Clin. Oncol. 2023, 20, 543. 37280366 10.1038/s41571-023-00782-x

[30] S. Jiang , H. Li , L. Zhang , W. Mu , Y. Zhang , T. Chen , J. Wu , H. Tang , S. Zheng , Y. Liu , Y. Wu , X. Luo , Y. Xie , J. Ren , Nucleic Acids Res. 2024, 53, D1670. 10.1093/nar/gkae973 PMC11701665 39470721

