# Supplementary material for: Radiotherapy‐Associated Cellular Senescence and EMT Alterations Contribute to Distinct Disease Relapse Patterns in Locally Advanced Cervical Cancer
Source: Adv Sci (Weinh). 2025 Feb 4;12(12):2412574. doi: 10.1002/advs.202412574 (PMC11948074; doi:10.1002/advs.202412574)
Supplement: Supplementary file 1 — Supporting Information [file ADVS-12-2412574-s001.pdf]

ADVANCED SCIENCE
Open Access

Supporting Information

for *Adv. Sci.*, DOI 10.1002/advs.202412574

Radiotherapy-Associated Cellular Senescence and EMT Alterations Contribute to Distinct Disease Relapse Patterns in Locally Advanced Cervical Cancer

Lei Zhang, Jun Ma, Jun Zhang, Minjie Hu, Jinlin Cheng, Bin Hu, Junjun Zhou, Di Zhou, Yongrui Bai, Xiumei Ma, Jianming Tang*, Haiyan Chen* and Ying Jing**

Supporting Information

Radiotherapy-Associated Cellular Senescence and EMT Alterations Contribute to Distinct Disease Relapse Patterns in Locally Advanced Cervical Cancer

Lei Zhang [1, †], Jun Ma [2, †], Jun Zhang[3, 4], Minjie Hu[5], Jinlin Cheng[6], Bin Hu[1], Junjun Zhou[1], Di Zhou[1], Yongrui Bai[1], Xiumei Ma[1, *], Jianming Tang [5, *], Haiyan Chen [1, *], Ying Jing [3, 4 *]

1 Department of Radiation Oncology, Renji Hospital, School of Medicine, Shanghai Jiao Tong University, Shanghai, 200127, China;
2 Eye Institute, Eye & ENT Hospital, Shanghai Medical College, Fudan University, Shanghai, 200031, China;
3 Center for Intelligent Medicine Research, Greater Bay Area Institute of Precision Medicine (Guangzhou), School of Life Sciences, Fudan University, Guangzhou, 511400, China;
4. State Key Laboratory of Genetic Engineering, Center for Evolutionary Biology, School of Life Sciences, Fudan University, Shanghai, 200438, China.
5 Department of Radiation Oncology, The First Hospital of Lanzhou University, Lanzhou University, Lanzhou, 730000, China
6. State Key Laboratory for Diagnosis and Treatment of Infectious Diseases, National Clinical Research Center for Infectious Diseases, National Medical Center for Infectious Diseases, Collaborative Innovation Center for Diagnosis and Treatment of Infectious Diseases, The First Affiliated Hospital, Zhejiang University School of Medicine, Hangzhou, Zhejiang, 310003, China

[†] These authors contributed equally to this work.

[*]Correspondence:

Ying Jing, Center for Intelligent Medicine Research, Greater Bay Area Institute of Precision Medicine (Guangzhou), School of Life Sciences, Fudan University, 2nd Nanjiang Road, Nansha District, Guangzhou, 511400, P. R. of China, Phone: 86-020-66333080. E-mail: jingying@ipm-gba.org.cn
Or Haiyan Chen, Department of Radiation Oncology, Renji Hospital, School of Medicine, Shanghai Jiao Tong University, No. 160 Pujian Road, Pudong New District, Shanghai, 200127, P. R. of China. Phone: 86-021-68383459. E-mail: chenhaiyan@renji.com
Or Jianming Tang, Department of Radiation Oncology, The First Hospital of Lanzhou University, Lanzhou University, No. 1 Donggang Road, No. 1 Donggang Road, Lanzhou, 730000, P.R. of China. Phone: 86-0931-8625222. E-mail: ldyy_tangjm@lzu.edu.cn
Or Xiumei Ma, Department of Radiation Oncology, Renji Hospital, School of Medicine, Shanghai Jiao Tong University, No. 160 Pujian Road, Pudong New District, Shanghai, 200127, P. R. of China. Phone: 86-021-68383459. E-mail: maxiumei@renji.com

Figure S1

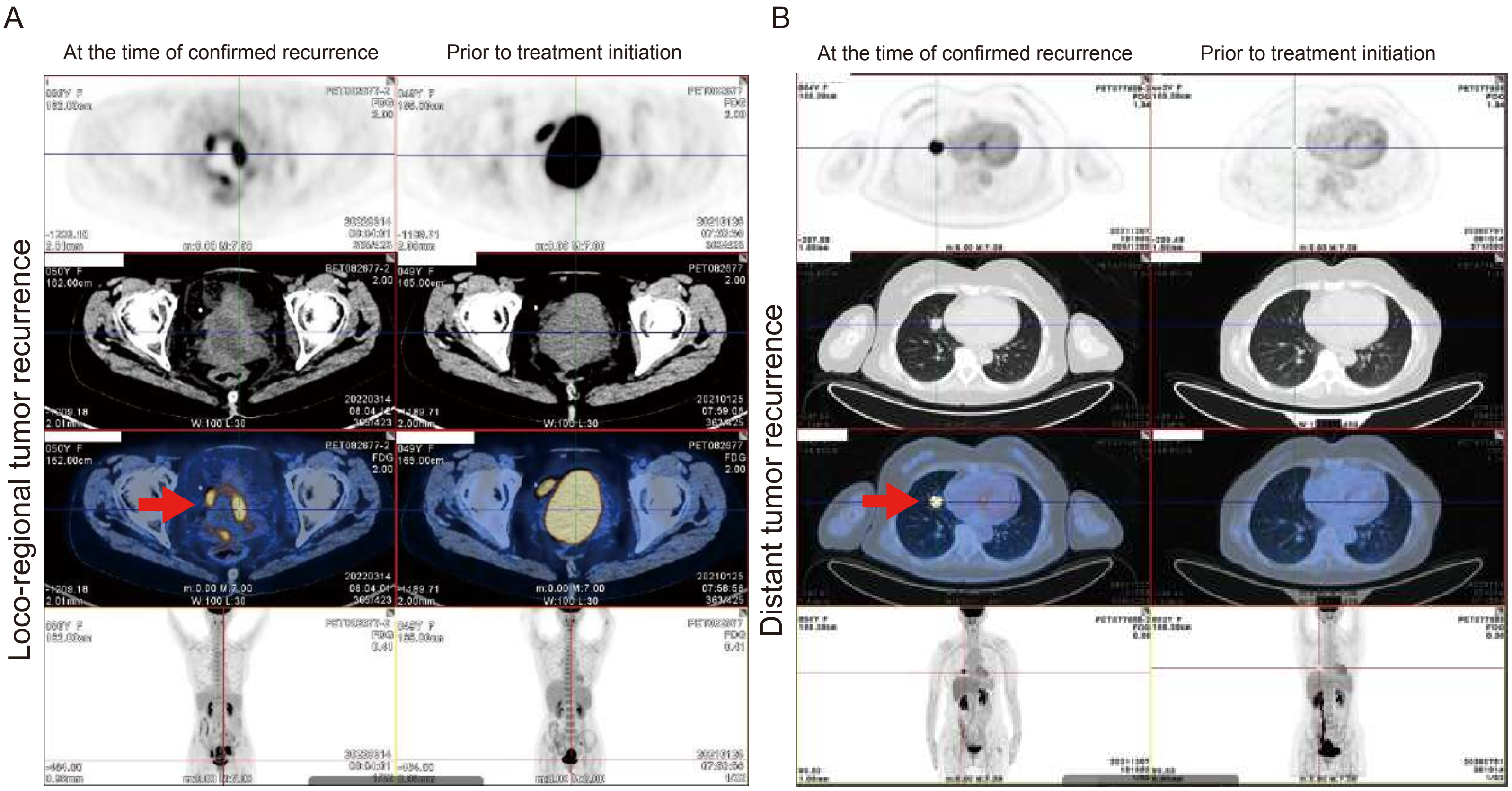

Figure S1. Representative images of LACC patients with locoregional or distant tumor recurrence.
A, Image scans of a 49-year-old patient with locoregional tumor recurrence following radiotherapy. The left column shows images taken at the time of tumor recurrence, while the right column shows images taken before radiotherapy. B, Image scans of a 54-year-old patient with distant tumor recurrence following radiotherapy. The left column shows images taken at the time of tumor recurrence, while the right column shows images taken before radiotherapy. Red arrows indicate the locations of the lesions. From top to bottom, the images display the ^{18}F-FDG scan, the CT scan, the merged ^{18}F-FDG and CT scans, and the whole-body view.

Figure S2

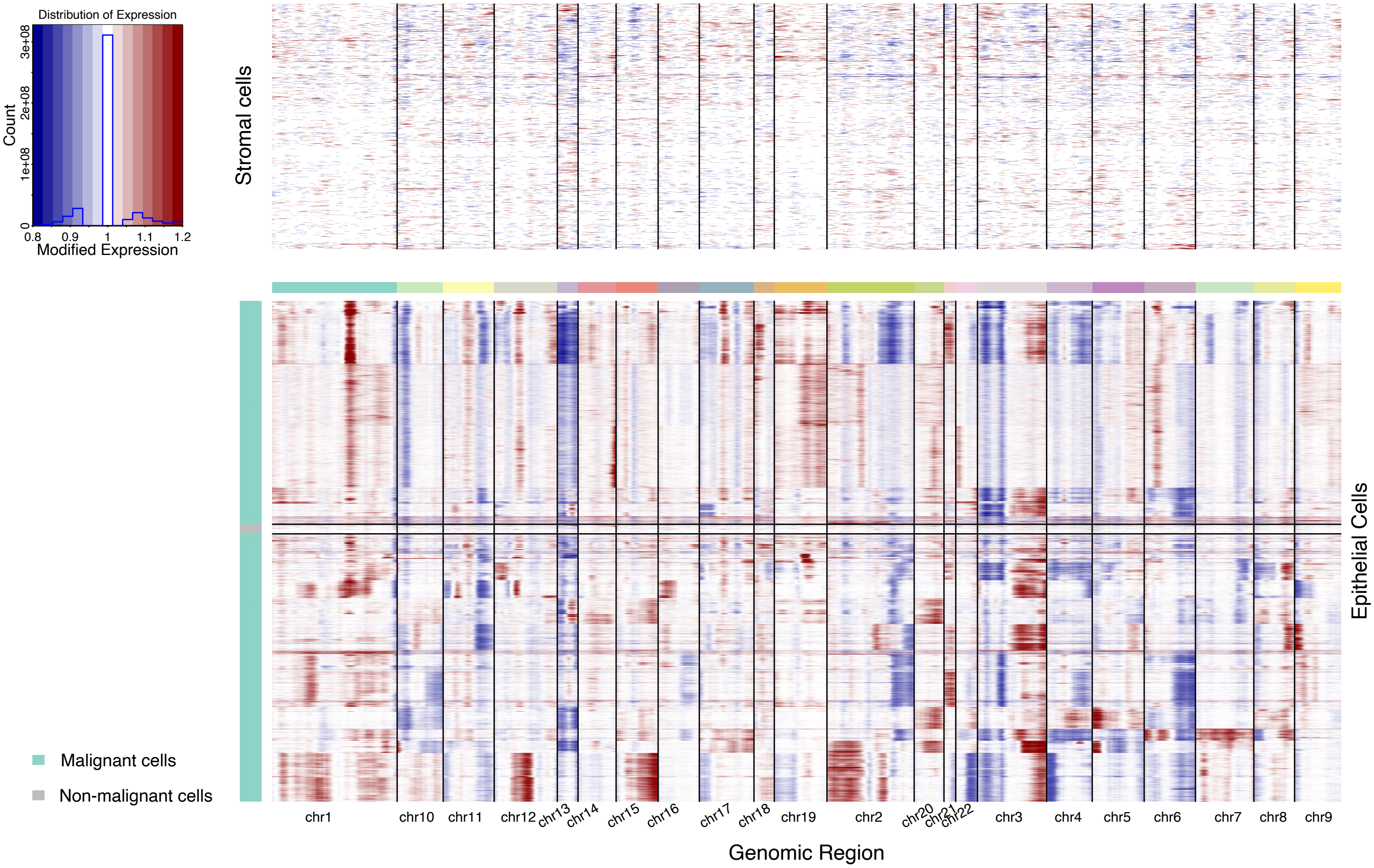

Figure S2. The landscape of inferred large-scale CNVs for all of the epithelial cells.

Figure S3

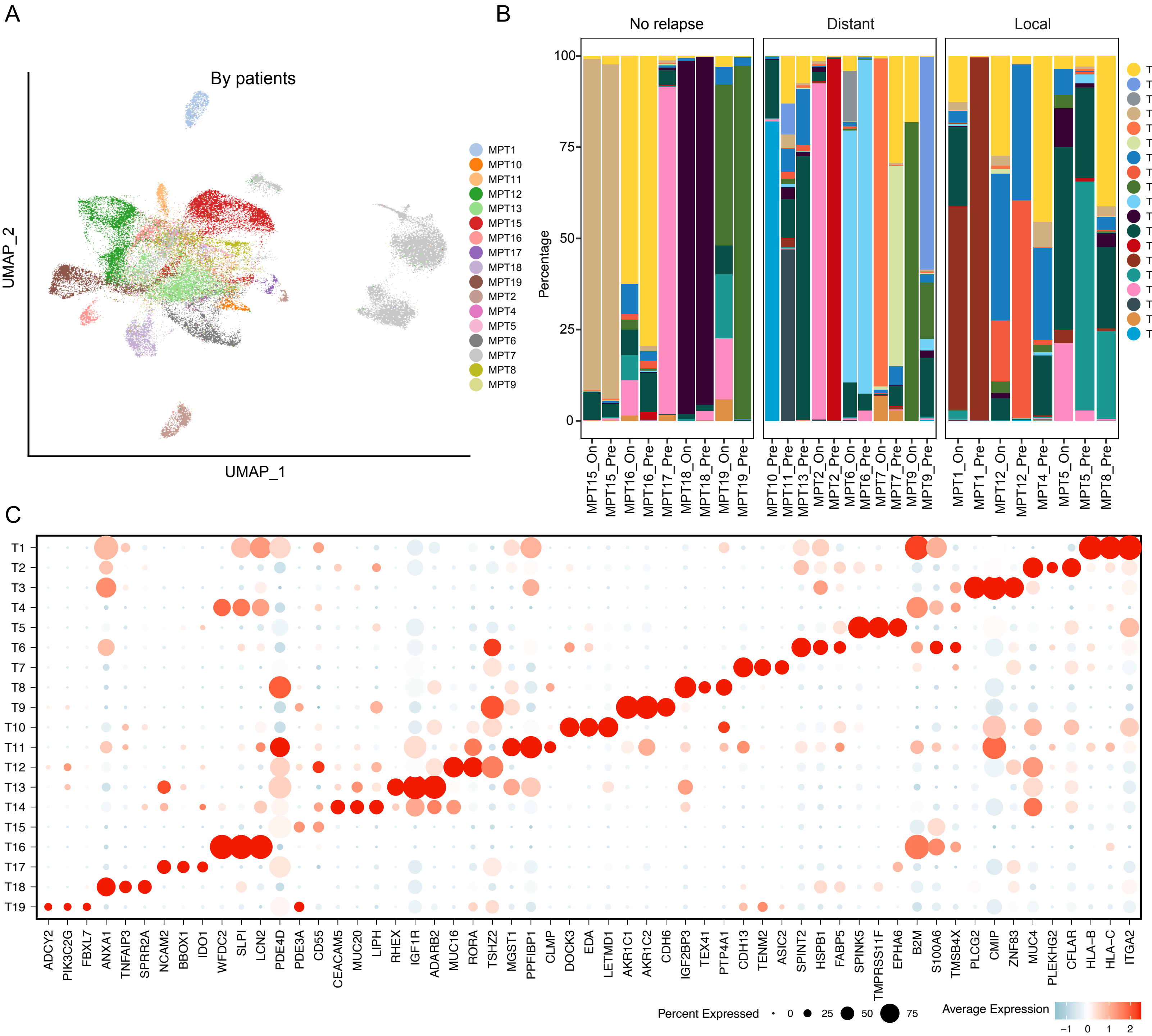

Figure S3. The cluster information for malignant cells. A, Uniform manifold approximation and projection plot of malignant cells from all the tumor samples. Each dot corresponds to a single nucleus, color coded by samples. B, The malignant cell composition in each tumor sample. The percentages (y axis) of the malignant cell subclusters in all the patients with different disease relapse patterns. C, Marker gene expression in the malignant cell subclusters. The bubble size indicates the percentage of cells in one subcluster expressing a gene and color intensity indicates the average scaled gene expression.

Figure S4

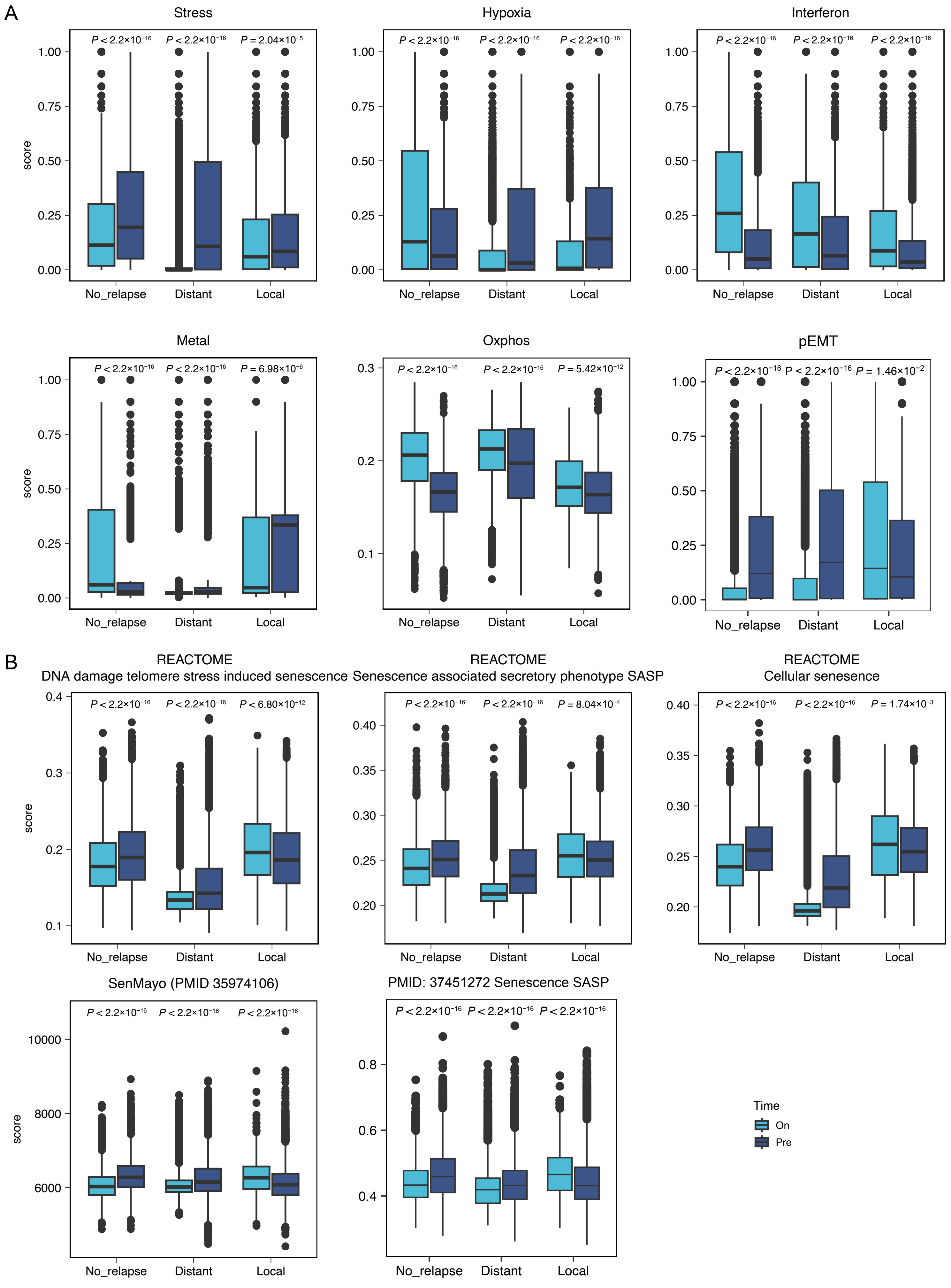
A
Stress
P < 2.2×10⁻¹⁶ P < 2.2×10⁻¹⁶ P = 2.04×10⁻⁵
Hypoxia
P < 2.2×10⁻¹⁶ P < 2.2×10⁻¹⁶ P < 2.2×10⁻¹⁶
Interferon
P < 2.2×10⁻¹⁶ P < 2.2×10⁻¹⁶ P < 2.2×10⁻¹⁶
Metal
P < 2.2×10⁻¹⁶ P < 2.2×10⁻¹⁶ P = 6.98×10⁻⁶
Oxphos
P < 2.2×10⁻¹⁶ P < 2.2×10⁻¹⁶ P = 5.42×10⁻¹²
pEMT
P < 2.2×10⁻¹⁶ P < 2.2×10⁻¹⁶ P = 1.46×10⁻²
score
No_relapse
Distant
Local
B
REACTOME
DNA damage telomere stress induced senescence
P < 2.2×10⁻¹⁶ P < 2.2×10⁻¹⁶ P < 6.80×10⁻¹²
REACTOME
Senescence associated secretory phenotype SASP
P < 2.2×10⁻¹⁶ P < 2.2×10⁻¹⁶ P = 8.04×10⁻⁴
REACTOME
Cellular senesence
P < 2.2×10⁻¹⁶ P < 2.2×10⁻¹⁶ P = 1.74×10⁻³
SenMayo (PMID 35974106)
P < 2.2×10⁻¹⁶ P < 2.2×10⁻¹⁶ P < 2.2×10⁻¹⁶
PMID: 37451272 Senescence SASP
P < 2.2×10⁻¹⁶ P < 2.2×10⁻¹⁶ P < 2.2×10⁻¹⁶
Time
On
Pre

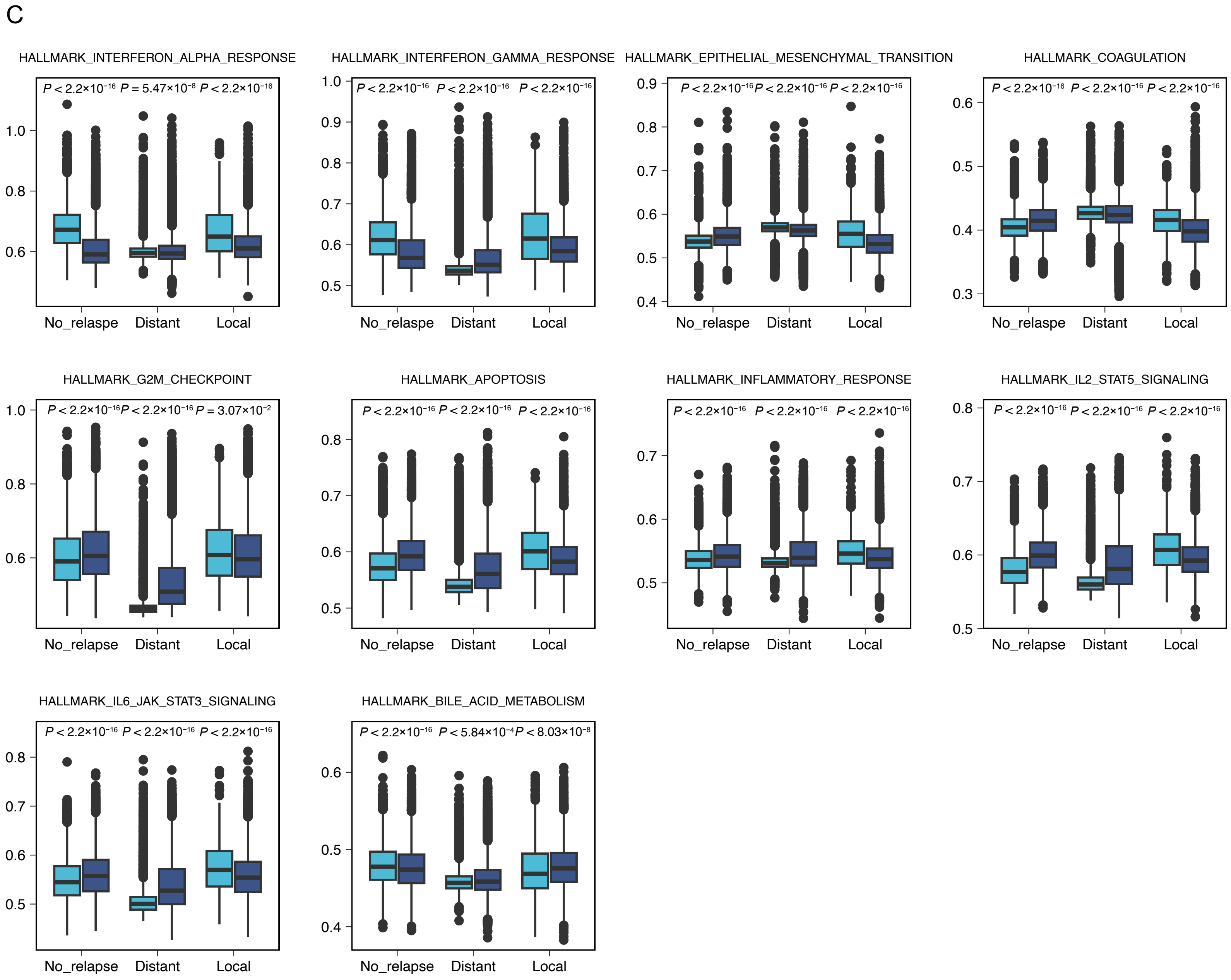

Figure S4. Comparisons of the expression levels of cancer cell state (A), cellular senescence (B) and hallmark gene sets (C) in malignant cells from pre- or on-treatment tumors in the three disease relapse groups. The center line indicates the median value, the lower and upper hinges represent the 25th and 75th percentiles, respectively, and the whiskers denoting the 1.5 interquartile range. The P values were calculated by two-sided Wilcoxon rank-sum test.

Figure S5

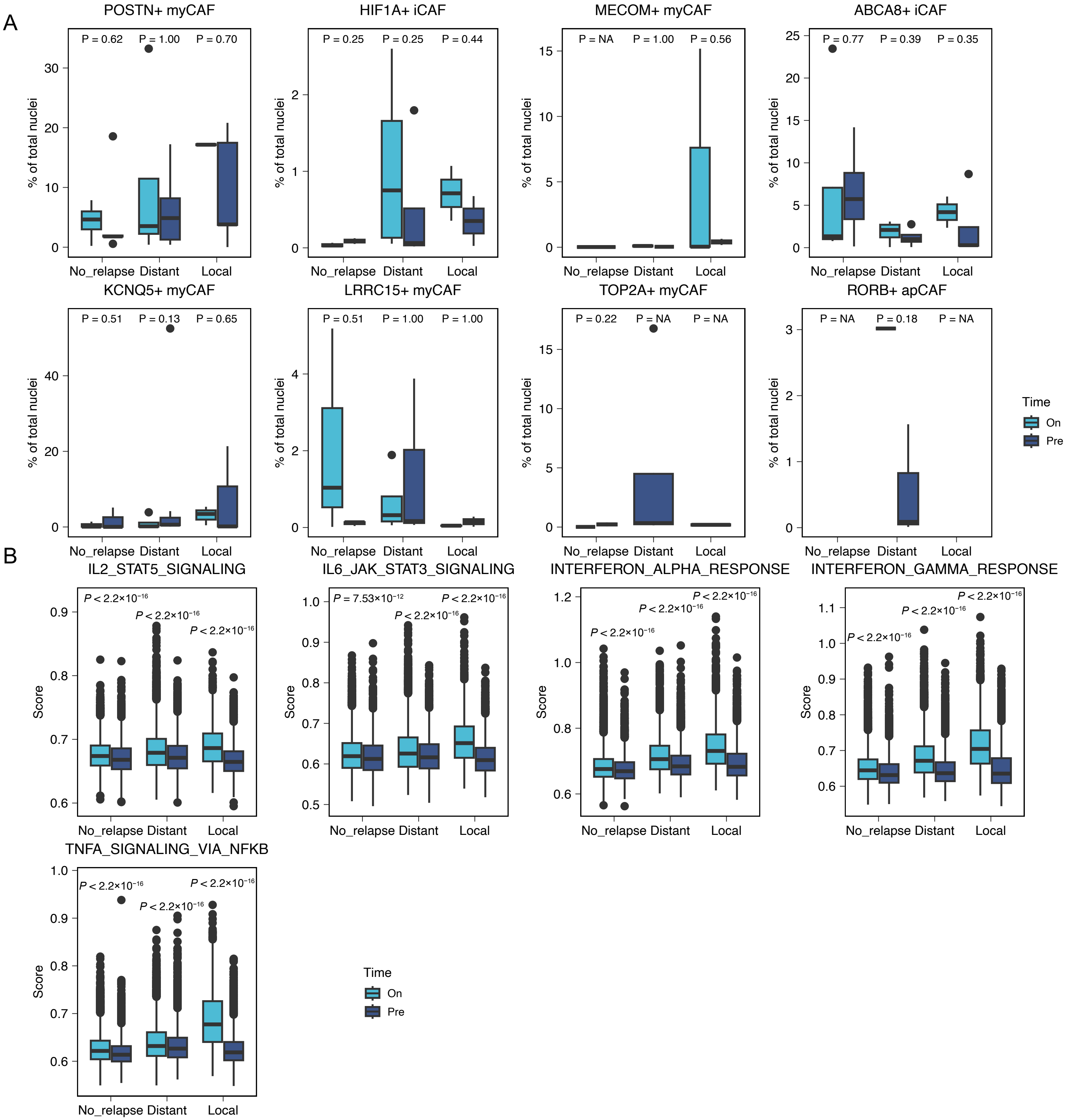

Figure S5. Comparing the proportion and expression of fibroblast cells from patients with different disease relapse patterns. A, Boxplots showing the proportion of fibroblast subclusters in pre- and on-treatment tumors in the different disease relapse statuses. The center line indicates the median value, the lower and upper hinges represent the 25th and 75th percentiles, respectively, and the whiskers denoting the 1.5 interquartile range. The P values were calculated by two-sided Wilcoxon rank-sum test. B, Boxplots showing the expression levels of the hallmark gene sets in fibroblasts from pre- and on-treatment tumor across different disease relapse statuses. The center line indicates the median value, the lower and upper hinges represent the 25th and 75th percentiles, respectively, and the whiskers denoting the 1.5 interquartile range. The P values were calculated by two-sided Wilcoxon rank-sum test.

Figure S6

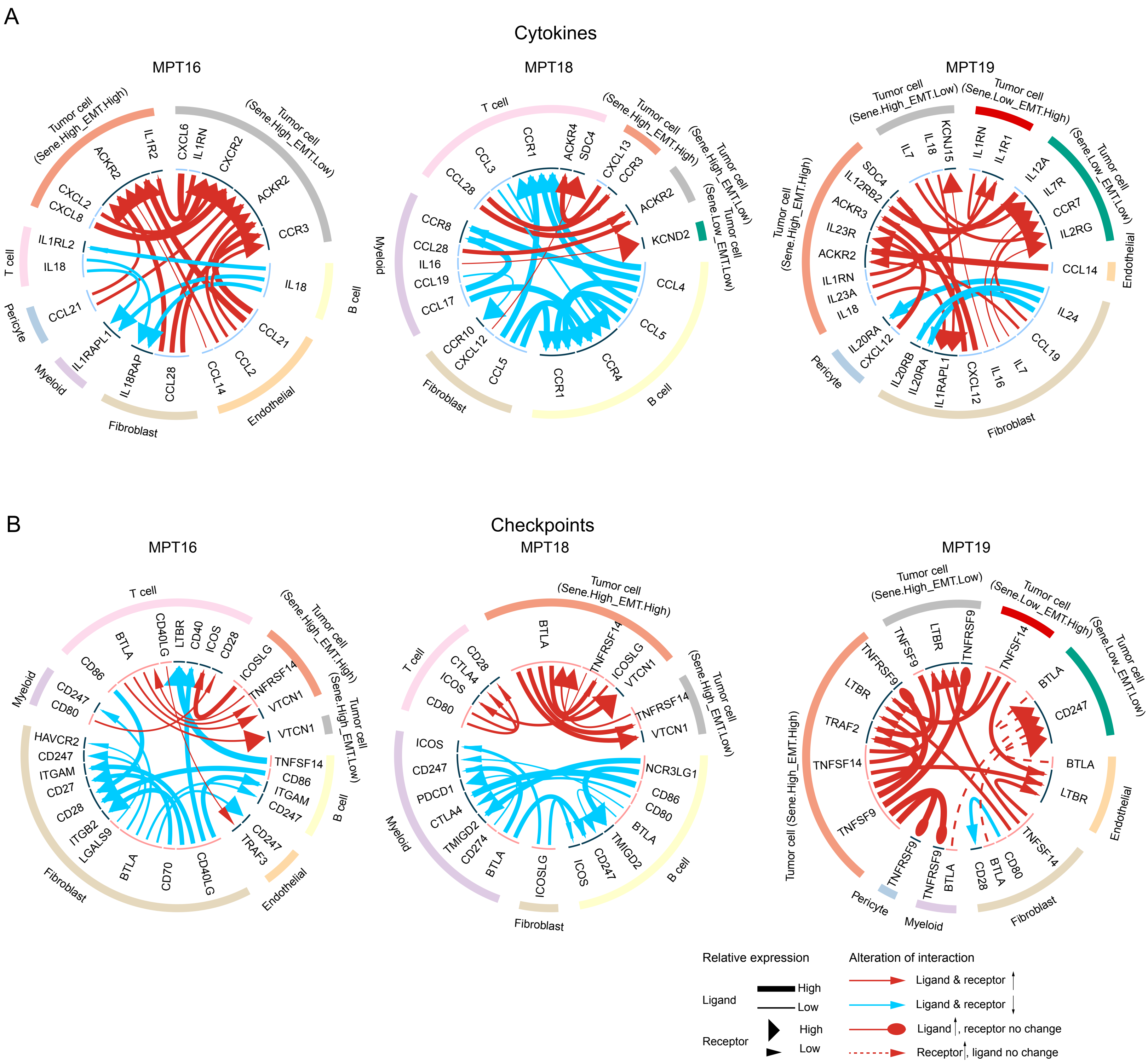

Figure S6. The enriched ligand–receptor cell–cell interactions in the major cell types in patients without disease relapse. A-B, Representative circos plots showing details of the top 20 differentially regulated cytokines (A) and checkpoints (B) ligand–receptor pairs when comparing on- versus pre-treatment tumors in the indicated patient. The lines are colored according their alterations. The size of the lines indicates the relative expression of ligands. The size of the arrows indicates the relative expression of receptors.

Figure S7

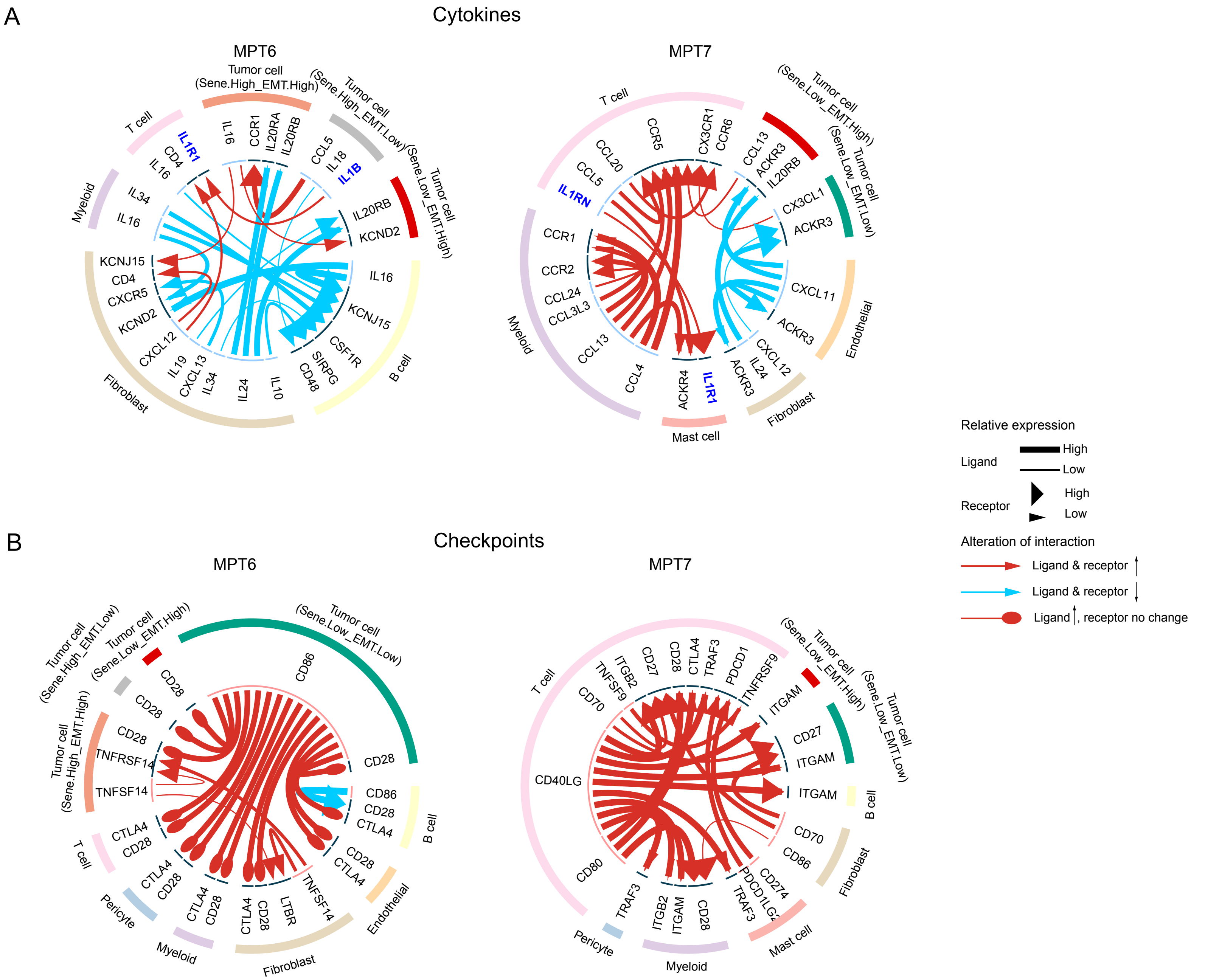

Figure S7. The enriched ligand–receptor cell–cell interactions in the major cell types in patients with distant disease relapse. A-B, Representative circos plots showing details of the top 20 differentially regulated cytokines (A) and checkpoints (B) ligand–receptor pairs when comparing on- versus pre-treatment tumors in the indicated patient. The lines are colored according their alterations. The size of the lines indicates the relative expression of ligands. The size of the arrows indicates the relative expression of receptors.

Figure S8

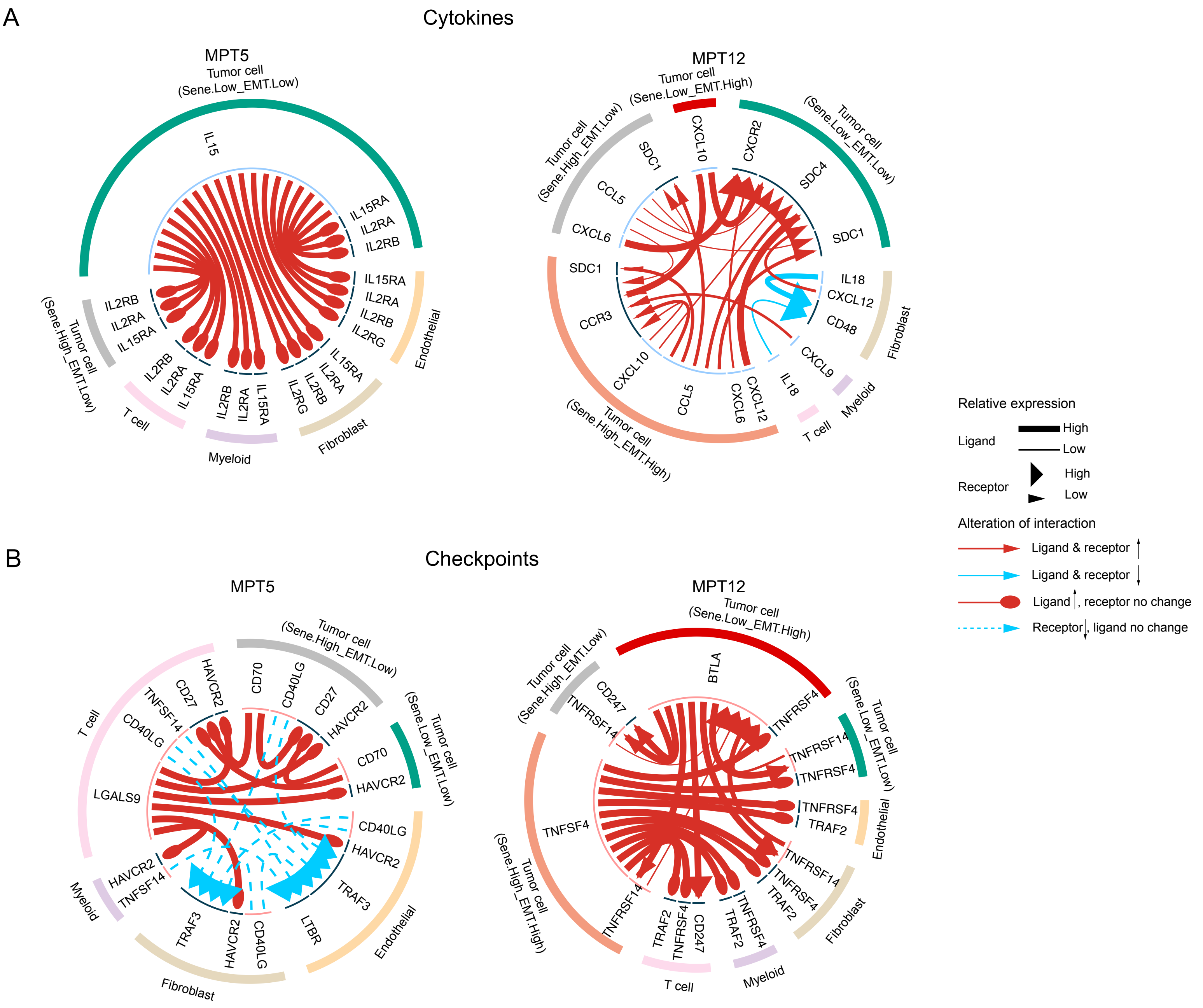

Figure S8. The enriched ligand–receptor cell–cell interactions in all the major cell types in patients with local disease relapse. A-B, Representative circos plots showing details of the top 20 differentially regulated cytokines (A) and checkpoints (B) ligand–receptor pairs when comparing on- versus pre-treatment tumors in the indicated patient. The lines are colored according to their alterations. The size of the lines indicates the relative expression of ligands. The size of the arrows indicates the relative expression of receptors.

Figure S9

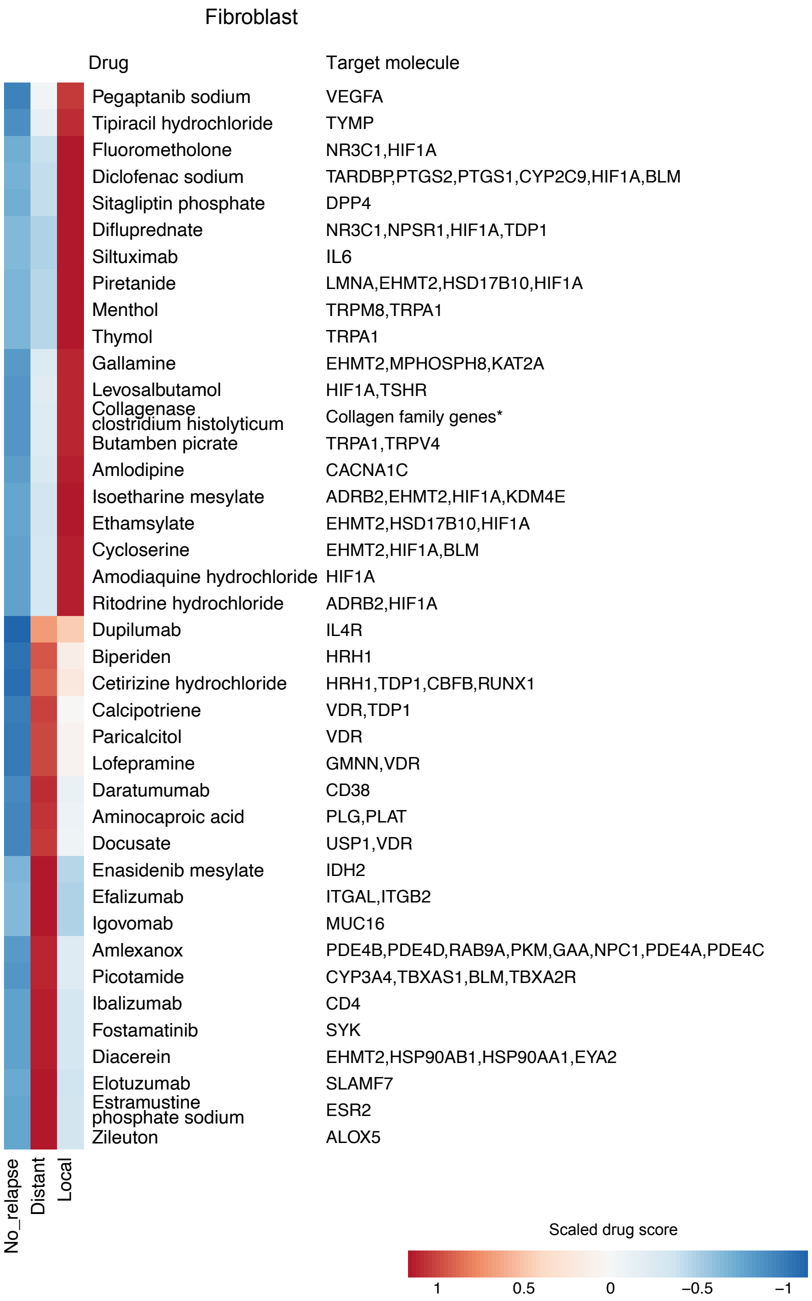

Figure S9. A heatmap of the significantly elevated drug scores in fibroblasts from patients with local and distant tumor recurrence compared with those without disease relapse (two-sided Wilcoxon test, P value < 0.05). The top 20 drugs for each group are illustrated based on fold changes. The colors of the heatmap represent z-score scaled values for each drug.

Figure S10

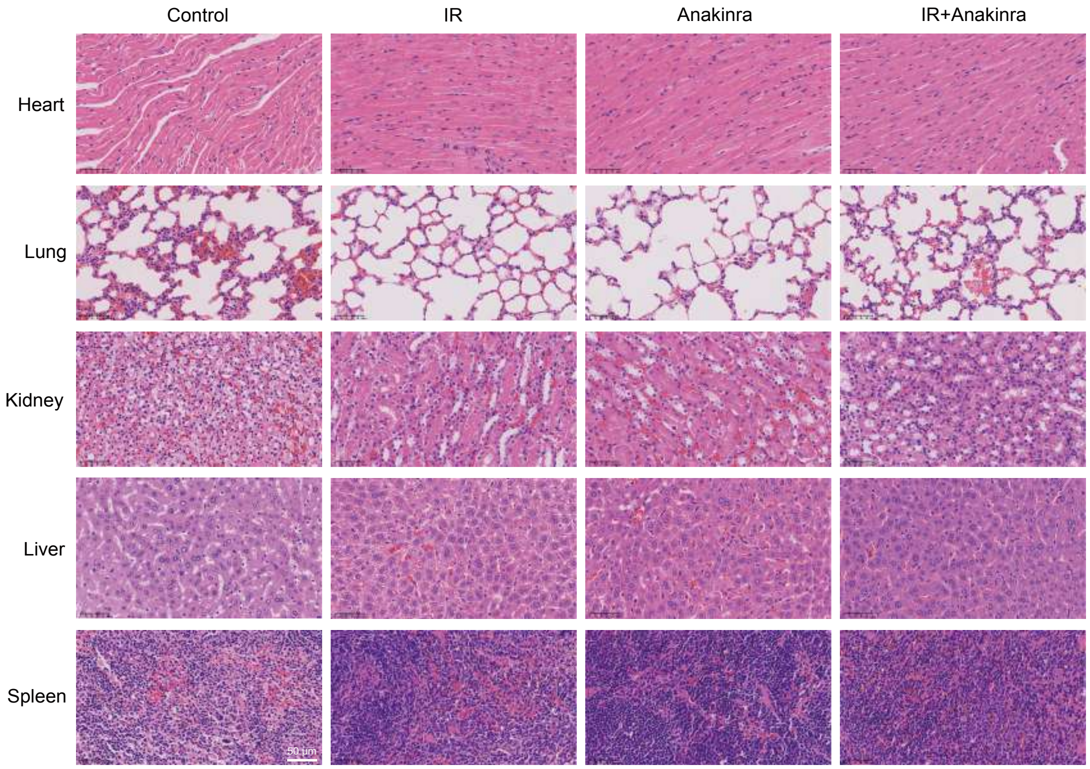

Figure S10. Evaluation of toxicities in BALB/c nude mice across different treatment groups. Representative hematoxylin and eosin (H & E) staining images of major organs from BALB/c nude mice. Tissues were collected and stained at the end of each treatment group (n = 5) and stained by H & E. Scale bar = 50 μm.

Figure S11

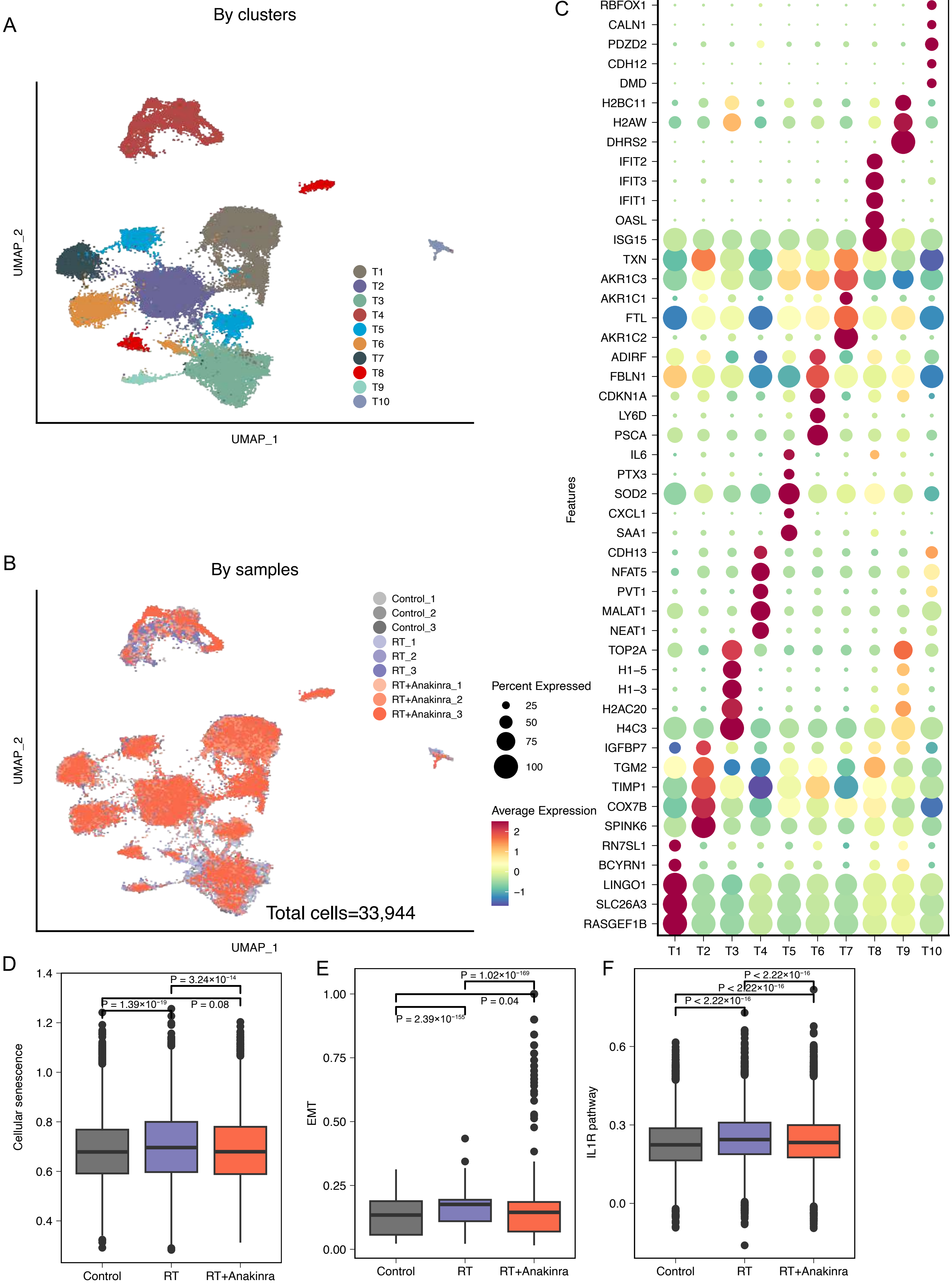
A
By clusters
UMAP_2
UMAP_1
T1
T2
T3
T4
T5
T6
T7
T8
T9
T10
B
By samples
UMAP_2
UMAP_1
Control_1
Control_2
Control_3
RT_1
RT_2
RT_3
RT+Anakinra_1
RT+Anakinra_2
RT+Anakinra_3
Total cells=33,944
C
Features
RBFOX1
CALN1
PDZD2
CDH12
DMD
H2BC11
H2AW
DHRS2
IFIT2
IFIT3
IFIT1
OASL
ISG15
TXN
AKR1C3
AKR1C1
FTL
AKR1C2
ADIRF
FBLN1
CDKN1A
LY6D
PSCA
IL6
PTX3
SOD2
CXCL1
SAA1
CDH13
NFAT5
PVT1
MALAT1
NEAT1
TOP2A
H1−5
H1−3
H2AC20
H4C3
IGFBP7
TGM2
TIMP1
COX7B
SPINK6
RN7SL1
BCYRN1
LINGO1
SLC26A3
RASGEF1B
T1 T2 T3 T4 T5 T6 T7 T8 T9 T10
Percent Expressed
25
50
75
100
Average Expression
2
1
0
−1
D
Cellular senescence
1.4
1.2
1.0
0.8
0.6
0.4
P = 3.24×10−14
P = 1.39×10−19
P = 0.08
Control
RT
RT+Anakinra
E
EMT
1.00
0.75
0.50
0.25
0.00
P = 1.02×10−169
P = 0.04
P = 2.39×10−155
Control
RT
RT+Anakinra
F
IL1R pathway
0.6
0.3
0.0
P < 2.22×10−16
P < 2.22×10−16
P < 2.22×10−16
Control
RT
RT+Anakinra

Figure S11. Cluster information for all the cells across different treatment groups. A-B, Uniform manifold approximation and projection plots showing the distribution of tumor clusters and cells from the samples obtained. C, Marker gene expression in the defined tumor cell clusters. The bubble size indicates the percentage of cells in a cluster expressing a gene and the color intensity indicates the average scaled-up gene expression levels. D-F, Comparisons of the expression levels of cellular senescence (D), EMT module score (E) and IL1R pathway (F) in tumor cells from different treatment groups. The center line indicates the median value, the lower and upper hinges represent the 25th and 75th percentiles, respectively, and the whiskers denoting the 1.5 interquartile range. The P values were calculated by two-sided Wilcoxon rank-sum test.
